# Data-science driven autonomous process optimization

**DOI:** 10.1038/s42004-021-00550-x

**Published:** 2021-08-02

**Authors:** Melodie Christensen, Lars P. E. Yunker, Folarin Adedeji, Florian Häse, Loïc M. Roch, Tobias Gensch, Gabriel dos Passos Gomes, Tara Zepel, Matthew S. Sigman, Alán Aspuru-Guzik, Jason E. Hein

**Affiliations:** 1 Department of Chemistry, University of British Columbia, Vancouver, BC Canada; 2 Department of Process Research and Development, Merck & Co., Inc., Rahway, NJ USA; 3 Department of Chemistry and Chemical Biology, Harvard University, Cambridge, MA USA; 4 Department of Chemistry, University of Toronto, Toronto, ON Canada; 5 Department of Computer Science, University of Toronto, Toronto, ON Canada; 6 Vector Institute for Artificial Intelligence, Toronto, ON Canada; 7 ChemOS Sàrl, Lausanne, Vaud Switzerland; 8 Department of Chemistry, University of Utah, Salt Lake City, UT USA; 9 Canadian Institute for Advanced Research, Toronto, ON Canada

**Keywords:** Process chemistry, Catalytic mechanisms, Synthetic chemistry methodology

## Abstract

Autonomous process optimization involves the human intervention-free exploration of a range process parameters to improve responses such as product yield and selectivity. Utilizing off-the-shelf components, we develop a closed-loop system for carrying out parallel autonomous process optimization experiments in batch. Upon implementation of our system in the optimization of a stereoselective Suzuki-Miyaura coupling, we find that the definition of a set of meaningful, broad, and unbiased process parameters is the most critical aspect of successful optimization. Importantly, we discern that phosphine ligand, a categorical parameter, is vital to determination of the reaction outcome. To date, categorical parameter selection has relied on chemical intuition, potentially introducing bias into the experimental design. In seeking a systematic method for selecting a diverse set of phosphine ligands, we develop a strategy that leverages computed molecular feature clustering. The resulting optimization uncovers conditions to selectively access the desired product isomer in high yield.

## Abstract (web summary)

An automated closed-loop system optimizes a stereoselective Suzuki-Miyaura reaction using a machine learning algorithm that incorporates unbiased and categorical process parameters.

## Introduction

Recent advancements in computer science and automation technologies have led to the emergence of autonomous chemistry systems designed to generate and test hypotheses without the need for constant researcher intervention^1–4^. Such systems typically involve three key components: (1) a machine learning (ML) algorithm for hypothesis generation, (2) a robotic system for experimental execution, and (3) an online analytics platform for performance evaluation. The system then executes a workflow with little to no human intervention. The level of human intervention varies based on the degree of hardware and software integration among the key components. In a fully autonomous system, a “closed loop” is achieved, where the scientist can define the search space, and then press “go”. Closed-loop applications have ranged from biologically active compound discovery^5,6^ to materials development^7–9^, novel reaction scouting^10–12^, and process optimization in flow reactors^13–23^.

Autonomous process optimization involves the human-intervention-free exploration of a range of predefined process parameters to improve responses such as reaction yield, product selectivity, and catalyst turnover number. The definition of a set of meaningful, broad, and unbiased process parameters is arguably the most critical aspect of a successful optimization. Work to date has focused on the multivariate optimization of continuous parameters such as temperature, stoichiometry, and time; however, vital categorical parameters such as reagent, solvent, or catalyst have rarely been incorporated. In fact, leading examples involving continuous and categorical parameter combinations have been limited to fewer than eight catalysts or ten solvents^24–26^. Furthermore, in these examples, categorical parameter selection was guided through chemical intuition, potentially introducing an element of bias into the experimental design. Thus, in our view, categorical parameter selection in the context of autonomous process optimization remains an unsolved challenge. We envisioned developing a more systematic method for the selection of a broad and diverse set of categorical parameters to fully represent the chemical space, driving more effective optimization campaigns.

Successful optimization also hinges on the identification of suitable automation equipment capable of effective experimental execution and analysis. Current research focuses heavily on custom-built continuous and segmented flow-reactor systems outfitted with online analytics for experimental execution. While these state-of-the-art systems have enabled the rapid multivariate optimization of several processes, examples have still been limited to the sequential execution of fast, homogeneous reactions amenable to flow reactors. In this work, we expand the autonomous process optimization toolkit to include a broader set of reaction methodologies by integrating off-the-shelf robotic systems with online analytics to carry out parallel reaction loops in 96-well plates.

The final aspect of successful optimization is the selection of an effective ML algorithm. Recently, Bayesian optimization (BO) algorithms have gained traction in the in autonomous chemistry realm, leading to a number of successful optimization campaigns^27–30^. BO constructs a statistical approximation of an unknown experimental response surface based on existing measurements to propose parameter points with promising predicted performance. The statistical approximation is refined with each measurement, resulting in an increasingly accurate response surface model. The algorithm is configured to balance the exploitation of areas of promising performance with the exploration of areas of uncertainty to allow for the determination of a global optimum response^31^. One limitation of BO is that parameter point selection is typically sequential. The Phoenics^32^ and Gryffin^33^ algorithms developed by Häse et al. supplement fundamental concepts from BO with a data smoothing technique (kernel density estimation) to suggest parameter points for parallel experiments. Gryffin was developed specifically for the parallel optimization of categorical parameters. Given our interest in the autonomous optimization of categorical and continuous parameters in tandem through the execution of parallel batch reactions, the Phoenics and Gryffin optimization strategies were deemed ideal fits in our optimization workflow.

**Fig. 1:**
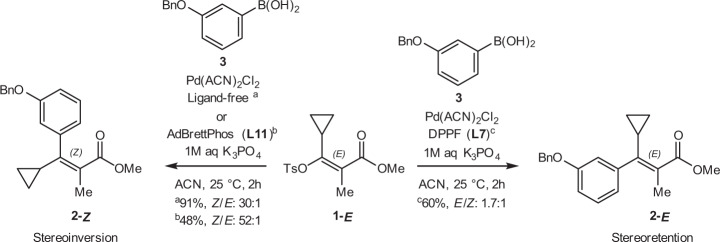
Phosphine ligand influence on a palladium-catalyzed stereoselective Suzuki–Miyaura coupling to generate the stereoinversion product (2-*Z*) or stereoretention product (2-*E*). ^a^Conditions: 10 µmol 1-*E*, 1 µmol 1,3,5-trimethoxybenzene, 20 µmol (3-(benzyloxy)phenyl)boronic acid 3, 0.4 µmol Pd(ACN)_2_Cl_2_, 30 µmol K_3_PO_4_ (0.5 M aq) in ACN (0.05 M), 2 h at 25 °C. ^b,c^Conditions: 10 µmol 1-*E*, 1 µmol 1,3,5-trimethoxybenzene, 11 µmol (3-(benzyloxy)phenyl)boronic acid 3, 0.2 µmol Pd(ACN)_2_Cl_2_, 0.4 µmol L, 30 µmol K_3_PO_4_ (0.5 M aq) in ACN (0.05 M), 2 h at 25 °C. Ligand structures are provided in Fig. 4. Tabulated results are provided in Supplementary Information Table SI-9.

We identified a stereoselective Suzuki–Miyaura cross-coupling reaction that would benefit from a tandem categorical and continuous parameter optimization (Fig. 1)^34,35^. Typically, Suzuki–Miyaura cross-couplings of vinyl halides or sulfonates proceed with retention of the olefin bond geometry^36^, but in this example, vinyl sulfonate 1-*E* undergoes significant stereoinversion under ligand-free and electron-rich dialkylbiaryl phosphine-mediated palladium catalysis to generate product 2-*Z*. In contrast, stereoinversion is partially suppressed under ferrocenyl bisphosphine-mediated palladium catalysis, facilitating a modest selectivity for product 2-*E*. Thus, stereoselectivity in this system appears to be influenced by both the phosphine ligand selection and stoichiometry^37–40^. Importantly, traditional phosphines preferred in Suzuki couplings, such as dialkylbiaryl phosphine ligands^41–44^, appear to facilitate the undesired steroinversion pathway. Finally, with reaction completion times on the order of two hours, this system is not amenable to flow reactors due to impractically long residence times.

Our goal was to improve the yield of stereoretention product 2-*E* through an autonomous optimization campaign by exploring a selection of phosphines and continuous process parameters in tandem. We employed a Chemspeed SWING robotic system for the experimental execution of parallel reaction loops in batch and employed the Phoenics and Gryffin algorithms for the proposal of parallel combinations continuous and categorical process parameter selections. Recognizing the impact of phosphine selection on the optimization outcome, we employed a variety of categorical parameter selection strategies, including chemical intuition and computed molecular descriptor clustering of 365 commercially available phosphines^45^. Here, we discuss the advantages and limitations of each phosphine selection strategy and their impacts on this challenging optimization problem.

## Results

### Establishing a closed-loop system

**Fig. 2:**
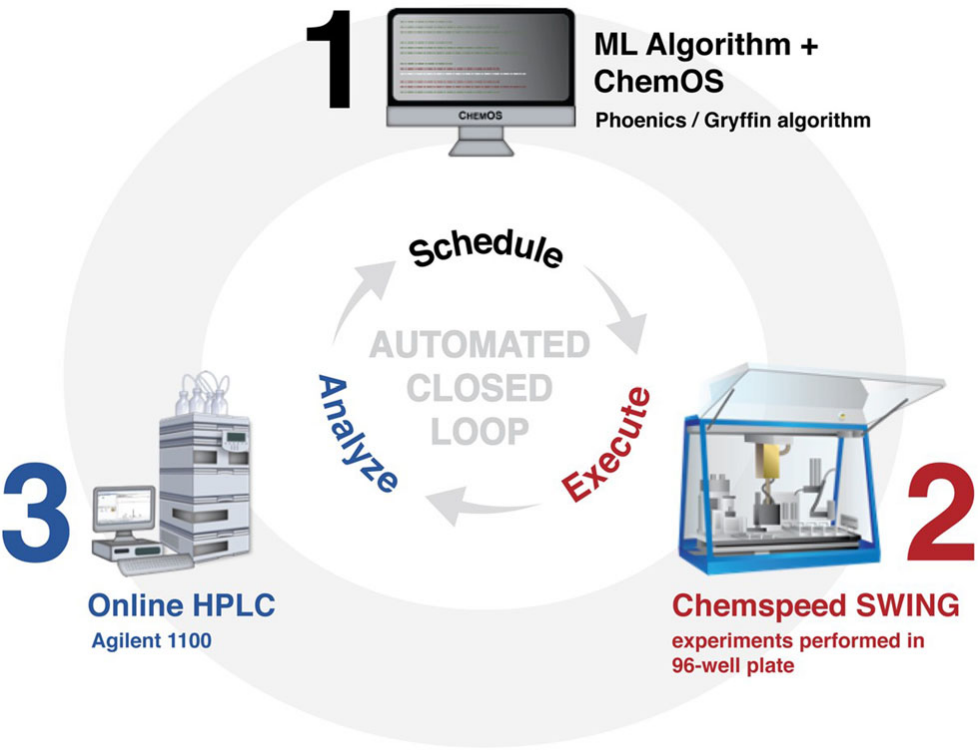
Closed-loop system for autonomous optimization in batch. The three main components to enable this closed loop include (1) ChemOS to coordinate experiments and data-driven approaches, (2) Chemspeed SWING for automated experimental setup, and (3) Agilent 1100 to characterize the experimental outcomes.

The establishment of a closed-loop system required the integration of three main components: (1) ChemOS^46^, the experimental scheduler for coordination of experiments proposed by the ML algorithms (Phoenics and Gryffin), (2) Chemspeed SWING, the robotic system for automated experimental setup, and (3) Agilent 1100, the HPLC-UV system for measurement of the experimental outcomes (Fig. 2). The only hardware customization required was the integration of the Agilent HPLC-UV system with the Chemspeed SWING robotic platform. This integration was accomplished through the installation of an HPLC valve on the Chemspeed robot deck and incorporation of relay switches for triggering chromatographic resolution and photodiode array detection^47^. The next step was to establish automated data flow from the experimental scheduler to the robot, and from the online analytical system back to the experimental scheduler for the ML algorithm to interpret results and propose subsequent experiments. In lieu of developing a complex application programming interface among the three software components (ChemOS, Chemspeed AutoSuite, and Agilent ChemStation), we opted to develop a lightweight Python framework for data transfer between these components. The script translated ChemOS parameter suggestions into stock mixture dispense volumes, calculated product assay yields from HPLC peak area ratios to an internal standard, and reported experimental measurements back to ChemOS.

### Defining the process parameters and optimization objectives

A set of categorical and continuous parameters was selected for potential impact on the reaction outcome, including phosphine ligand, phosphine to palladium ratio, palladium loading, arylboronic acid equivalents, and reaction temperature (Table 1). Upper and lower bound selection for each continuous parameter was guided by chemical intuition but also designed to be sufficiently broad to explore a diverse set of responses. Ligand set selections varied from 12 to up to 23 ligands, depending on the selection strategy. The ML algorithms (Phoenics and Gryffin) were configured to maximize the yield of the *E*-product, minimize the yield of the *Z*-product, minimize the palladium loading, and minimize the arylboronic acid equivalents, in that order (Table 1). Each objective was configured with a 10% relative threshold that would only consider the next objective once that threshold had been achieved. This multiobjective optimization, or Pareto optimization design, was made possible through the implementation of the scalarizing function Chimera^48^.

**Table 1:** Process parameters and optimization objectives.

Parameter	Type	Range	Unit
Phosphine ligand (P ligand)	Categorical	12–23	number of ligands
Phosphine to palladium ratio (P/Pd)	Continuous	0.5–4.0	ratio
Palladium loading (Pd mol%)	Continuous	1.0–5.0	mol%
Arylboronic acid equivalents (ArBA equiv)^a^	Continuous	1.0–2.0	equivalents
Reaction temperature (Rxn temp)	Continuous	10–40	°C

Response	Objective	Priority	Unit
*E*-product assay yield (*E*-PR AY)	Maximize	First	mol%
*Z*-product assay yield (*Z*-PR AY)	Minimize	Second	mol%
Palladium loading (Pd mol%)	Minimize	Third	mol%
Arylboronic acid equivalents (ArBA equiv)^a^	Minimize	Fourth	equivalents

^a^The arylboronic acid equivalents parameter and response were removed from the experimental design upon expanding the search space from 12 to 23 ligands.

### Designing the automation workflow and characterizing reproducibility

Firstly, the number of reactions for the effective evaluation of all selected ligands under a reasonable number of continuous parameter points was estimated to reside somewhere between 120 and 192. Secondly, conversion to product was estimated to complete within two hours under the median points of the defined continuous parameter ranges. Thus, if the maximum number of 192 consecutive reactions were carried out sequentially, the optimization campaign would take 16 days to complete. This lengthy duration would serve to obviate the benefits of autonomous optimization, and correspondingly, necessitated parallelization of the reactions. Therefore, the reactions were parallelized into loops of eight, allowing for a 192-reaction campaign to be completed within 24 loops over a more reasonable time period of four days. Conditions for the first loop of eight reactions were selected randomly by the ML algorithm. Subsequent conditions were determined autonomously by Phoenics and Gryffin as analytical results were returned, following a data-driven strategy. ChemOS, the experimental scheduler, parallelized the suggested reactions for the Chemspeed SWING system to dispense and initiate in 15-min intervals. Analytical samples were aliquoted and acquired at the endpoint of each reaction, also in 15-min intervals.

**Fig. 3:**
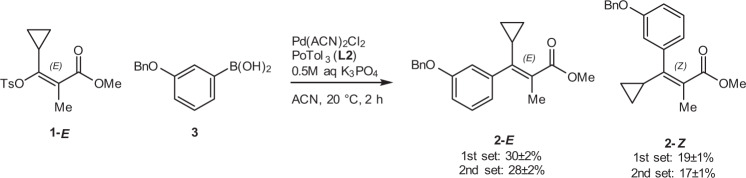
Standard experimental conditions for reproducibility testing. Conditions: 10 µmol 1-*E*, 1 µmol 1,3,5-trimethoxybenzene, 15 µmol (3-(benzyloxy)phenyl)boronic acid 3, 0.3 µmol Pd(ACN)_2_Cl_2_, 0.4 µmol L2, 30 µmol K_3_PO_4_ (0.5 M aq) in ACN (0.05 M), 2 h at 20 °C. Average yields for first set of replicates: 2-*E*: 30(±2)%; 2-*Z*: 19(±1)% and second set of replicates: 2-*E*: 28(±2)%; 2-*Z*: 17(±1)%. Tabulated results are provided in Supplementary Information Table SI-13.

In order to evaluate the reproducibility and system performance, two sets of eight standard experiments were carried out within in each reaction block of a 192-reaction optimization campaign. We determined that the standard deviation (SD) of measured 2-*E* and 2-*Z* yields fell between 1 and 2 mol% and relative SD ranged between 6 and 8% (Fig. 3). This level precision was determined to be acceptable for meaningful data interpretation.

### Trial runs and experimentally derived constraints for the optimization strategy

Trial runs unveiled two instrument constraints that necessitated further enhancements of the optimization strategy. The first constraint involved the need to fix the reaction temperature across each loop of eight reactions given that these reactions were designed to be carried out within the same reactor block and timeframe. In order to accommodate this constraint, the capabilities of the Phoenics and Gryffin optimization strategies were extended to facilitate optimization with process constraints following the idea of a previously introduced basic process-constrained BO algorithm (pc-BO(basic))^49^. This extension allowed the suggestion of a total of eight different experiments where the temperature was fixed across one loop. The second instrument constraint was the inability of the Chemspeed robot to execute submicroliter dispenses accurately; thus, the Python script was augmented to round calculated dispense volumes to the microliter level and update the suggested parameters with the executed parameters prior to returning analytical results to ChemOS. These enhancements allowed for the successful application of an algorithm in a constrained experimental setting.

### Autonomous optimization with ligands selected through chemical intuition

**Fig. 4:**
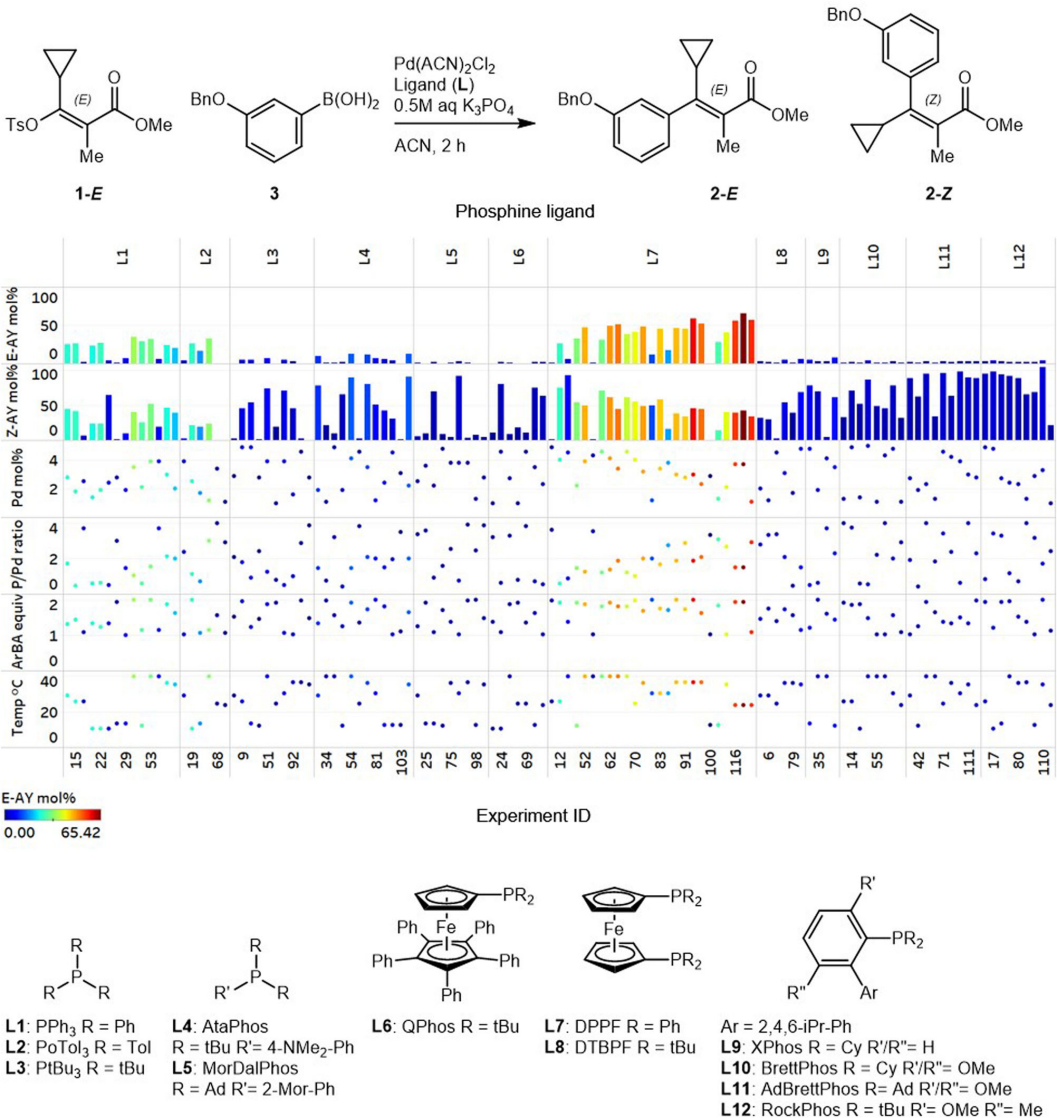
Parameters and results of optimization with ligands selected through chemical intuition in campaign 1. Conditions: 10 µmol 1-*E*, 1 µmol 1,3,5-trimethoxybenzene, 10–20 µmol (3-(benzyloxy)phenyl)boronic acid 3, 0.1–0.5 µmol Pd(ACN)_2_Cl_2_, 0.05–2 µmol L, 30 µmol K_3_PO_4_ (0.5 M aq) in ACN (0.05 M), 2 h at 10–40 °C. Tabulated results are provided in Supplementary Information Table SI-10.

Initial ligand selection was carried out through chemical intuition around phosphines with the potential to accelerate palladium-catalyzed cross-couplings^41–44^ (here, chemical intuition refers to insight arising from a combination of literature precedent and hands-on experience). Twelve ligands were selected, including trialkyl, triaryl, ferrocenyl, and dialkylbiaryl phosphines. These ligands were evaluated in combination with four continuous parameters, including phosphine to palladium ratio, palladium loading, arylboronic acid equivalents, and reaction temperature. The optimization campaign was carried out in 15 loops of eight reactions, totaling 120 autonomous iterations carried out over 60 h. Initial visualization of the two product yields highlights the strong propensity for stereoinversion under the majority of evaluated conditions, generating significant levels of product 2-*Z* with nine out of 12 ligands (L3–L6 and L8–L12, Fig. 4). Despite the demonstrated susceptibility for stereoinversion, the optimization ultimately resulted in conditions to access product 2-*E* in 65% yield and 1.6:1 *E*/*Z* selectivity upon 118 iterations, under Pd-L7 catalysis. Notably, the optimizer dedicated a higher number of iterations to L7 (DPPF), the top-performing ligand. Also, although initial results with L7 were not promising in iterations 11 (1% 2-*E*), 12 (26% 2-*E*), and 33 (6% 2-*E*), likely due to suboptimal phosphine to palladium ratios, the optimizer continued to sample this ligand exploratively, revealing a combination of continuous parameter points that resulted in the optimum conditions in iteration 118 (65% 2-*E*). Bearing in mind that the optimizer was not configured with any background knowledge around this reaction system except for the predefined process parameter ranges, arrival at this optimum is an impressive demonstration of the power of algorithmic optimization. It is also worth noting the algorithm’s repeated sampling of L11 and L12 despite poor performance under a number of parameter point selections. This behavior could potentially be attributed to explorative sampling or optimism bias in the algorithm’s predictions. Finally, although the optimum conditions aligned nicely with those identified previously in manual searches, improvement to the yield of 2-*E* was nominal (compared to 60% yield and 1.7:1 *E*/*Z* selectivity as shown in Fig. 1). This nominal improvement was attributed to ligand bias resulting from chemical intuition-based phosphine selection, thus, we embarked upon the exploration of a methodically selected set of ligands to access 2-*E* in even higher yield.

### Autonomous optimization with ligands selected through computed molecular features

**Fig. 5:**
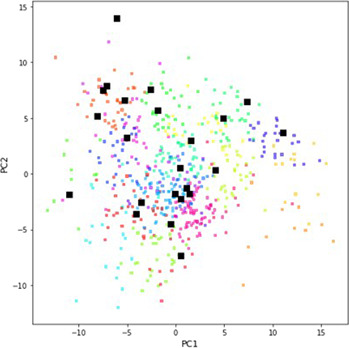
K-means clustering on the first four principal components of the molecular descriptor set for 365 commercial monodentate phosphines. The chemical space is represented by a two-dimensional plot of the first two principal components. Each cluster is represented by color and highlighted boxes indicate selected ligands. Selected ligand structures are provided in Fig. 6.

**Fig. 6:**
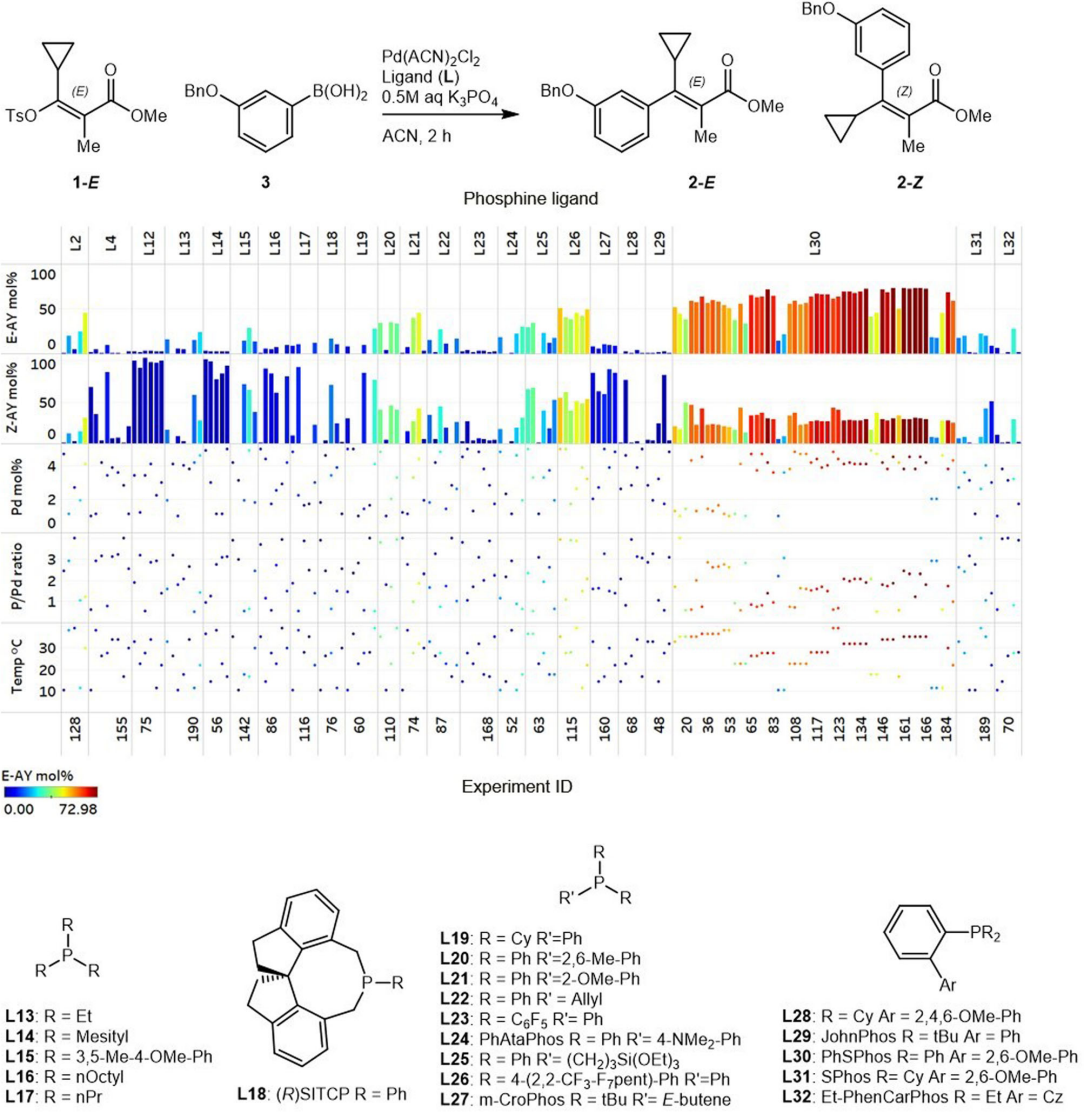
Parameters and results of optimization with ligands selected through descriptor clustering in campaign 2. Conditions: 10 µmol 1-*E*, 1 µmol 1,3,5-trimethoxybenzene, 15 µmol (3-(benzyloxy)phenyl)boronic acid 3, 0.1–0.5 µmol Pd(ACN)_2_Cl_2_, 0.05–2 µmol L, 30 µmol K_3_PO_4_ (0.5 M aq) in ACN (0.05 M), 2 h at 10–40 °C. Tabulated results are provided in Supplementary Information Table SI-11. An animated chart of *E*-product yield over time is provided as Supplementary Movie 2.

To this end, we sought a systematic method for the selection of a diverse set of phosphines for autonomous evaluation. A particularly attractive approach would leverage computed molecular features of phosphines as these have been applied to reaction optimization through predictive modeling^50–53^. In our current study, 365 commercially available monodentate phosphines were used to define the chemical space (the focus was limited to monodentate phosphines in order to more effectivity control the ligation state of palladium). For each phosphine, features were obtained by computing molecular properties for a representative set of conformers using DFT. Then, k-means clustering was carried out on the first four principal components of this descriptor set to divide the chemical space into 24 regions (Fig. 5, see Supplementary Information Descriptor Computation and Training Set Selection sections and Table SI-14 through 15 for details). A single compound was selected from each cluster for experimental evaluation based on additional considerations such as availability, price, and anticipated stability (Fig. 6). One cluster contained ligands that were deemed too challenging to implement due to low boiling points; therefore, candidates from this cluster were not included in the experimental design.

Investigation of a larger ligand set necessitated the expansion of the optimization campaign to 24 loops of eight reactions, totaling 192 autonomous iterations executed over 96 h (16 of which were designated as test reactions to assess reproducibility as shown in Fig. 4). In addition, the expanded ligand set also necessitated the removal of the arylboronic acid equivalents parameter and response from the experimental design. Initial visualization of the two product yields highlights the strong propensity for stereoinversion under the majority of evaluated conditions, generating significant levels of product 2-*Z* with 14 out of 23 ligands (L4, L12–L19, L23, L27–L29, and L31, Fig. 6). Despite this, the second campaign resulted in the identification of optimal conditions to access product 2-*E* in 73% yield and 2.5:1 *E*/*Z* selectivity upon 161 iterations, using L30 as the ligand. Given that the ML algorithm had no previous information to bias the search, and that ligand selection was unbiased, an improved yield of 73% highlights the potential of our novel optimization technology. As in the first campaign, the algorithm dedicated a significant portion of iterations to the top-performing ligand, previously L7 (DPPF), now L30 (PhSPhos). Additional high-performing ligands also fell under the triaryl phosphine category, with both electron-rich (L20, L21) and electron-poor (L26) triaryl phosphines proving effective. Surprisingly, ligands with significant structural similarity to L30 (PhSPhos), including L28 and L31 (SPhos), did not selectively yield 2-*E*, presumably due to the presence of electron-rich cyclohexyl substituents.

**Fig. 7:**
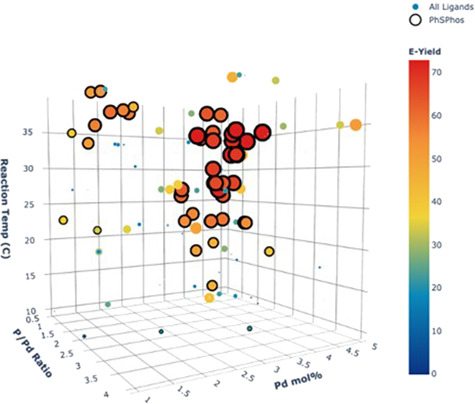
Three-dimensional plot of three continuous parameter selections color coded for 2-*E* yield in campaign 2. PhSPhos results are outlined; all other ligand results are not outlined.

A deeper look into the influence of the continuous parameters on the yield of product 2-*E* (Fig. 7) revealed that phosphine to palladium ratios within the center of the studied range provided optimal outcomes. As predicted, lower phosphine to palladium ratios resulted in an increase in 2-*Z* yields, potentially due to the presence of phosphine-free palladium, while higher phosphine to palladium ratios resulted in an overall lack of reactivity, likely due to blocking of coordination sites necessary for effective catalysis. Conversely, performing the reaction at temperatures near the upper bound of the evaluated range proved most effective in driving product formation. Finally, although higher palladium loadings resulted in improved product 2-*E* yields, the algorithm did not default to maximize the loading to drive up the yield. It appears that configuring the algorithm to minimize the palladium loading resulted in a constrained evaluation of this response and we were pleased to observe that the algorithm arrived at a more reasonable optimum of 3.8 mol% palladium loading.

**Fig. 8:**
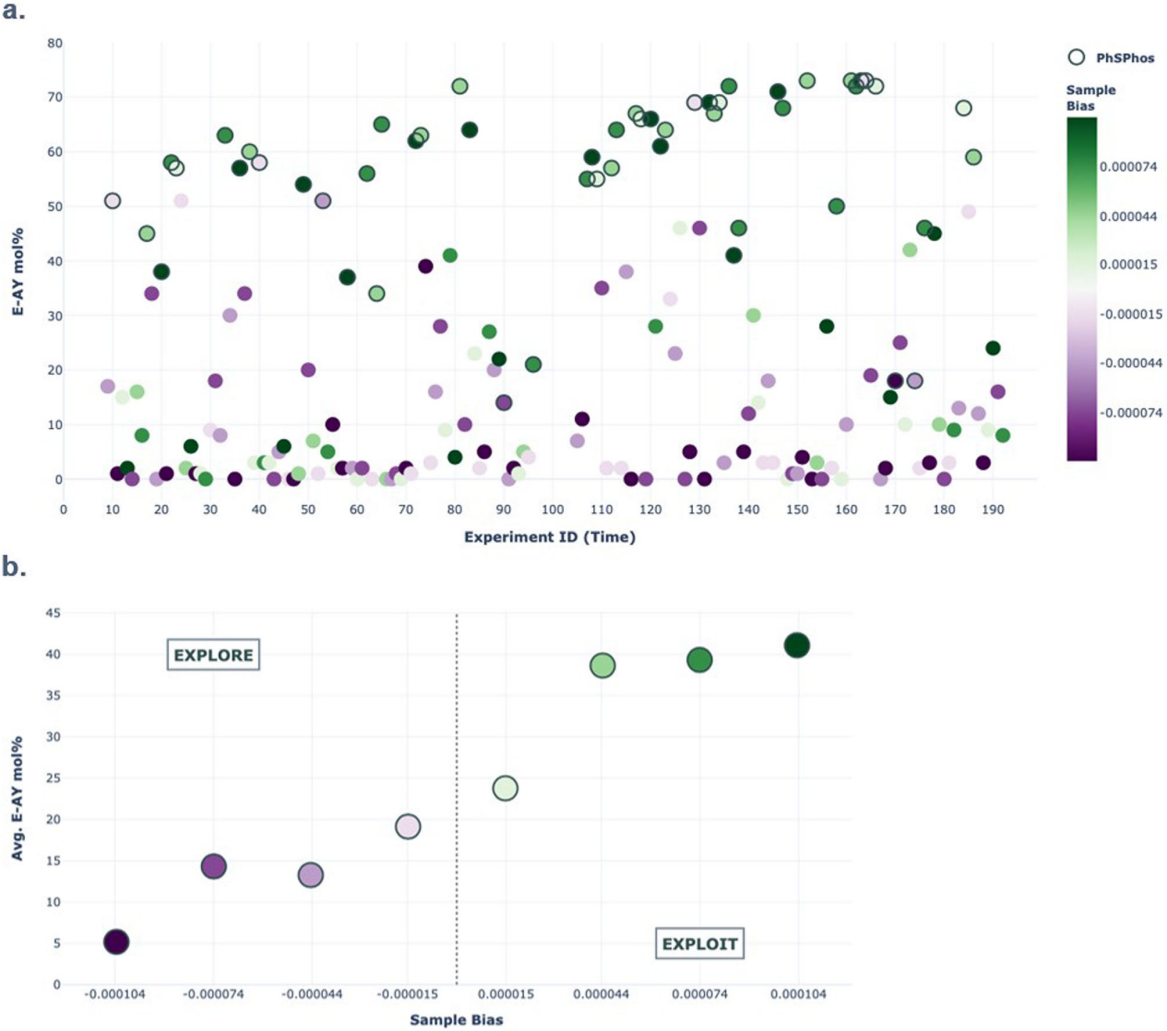
Two-dimensional plots of 2-*E* yield for each experiment ID and average 2-*E* yield for each sample bias value in campaign 2. **a** Green indicates positive, exploitative sample bias values, while purple indicates negative, explorative sample bias values. PhSPhos results are outlined; all other ligand results are not outlined. **b** Green indicates positive, exploitative sample bias values, while purple indicates negative, explorative sample bias values.

It can be challenging to evaluate the performance of a multivariate BO because much of the algorithm’s decision-making behavior occurs in a black box. One way to gain insight into the algorithm’s success is to look at the relationship between response variables, such as 2-*E* yield, and the algorithm’s tendency to explore or exploit. Sample bias is a value that measures the degree to which the algorithm was programmed to explore or exploit for any given experiment, and in this run, an array of eight evenly spaced sample bias values was selected, ranging from −0.000104 (highly explorative) to 0.000104 (highly exploitative). Plotting the yield of 2-*E* against experiment ID while color coding for sample bias reveals that a majority of the exploitative iterations focused on the top-performing ligand, PhSPhos (L30), leading to a significant yield improvement of 20% (Fig. 8a). Plotting the average yield of 2-*E* at each sample bias value shows that on average, negative explorative sample biases led to low 2-*E* yields, while positive exploitative sample biases led to high 2-*E* yields (Fig. 8b). This balanced strategy allowed for determination of the global optimum over the local optimum because high-performing ligands could have been missed under certain continuous parameter selections such as high phosphine to palladium ratio.

**Fig. 9:**
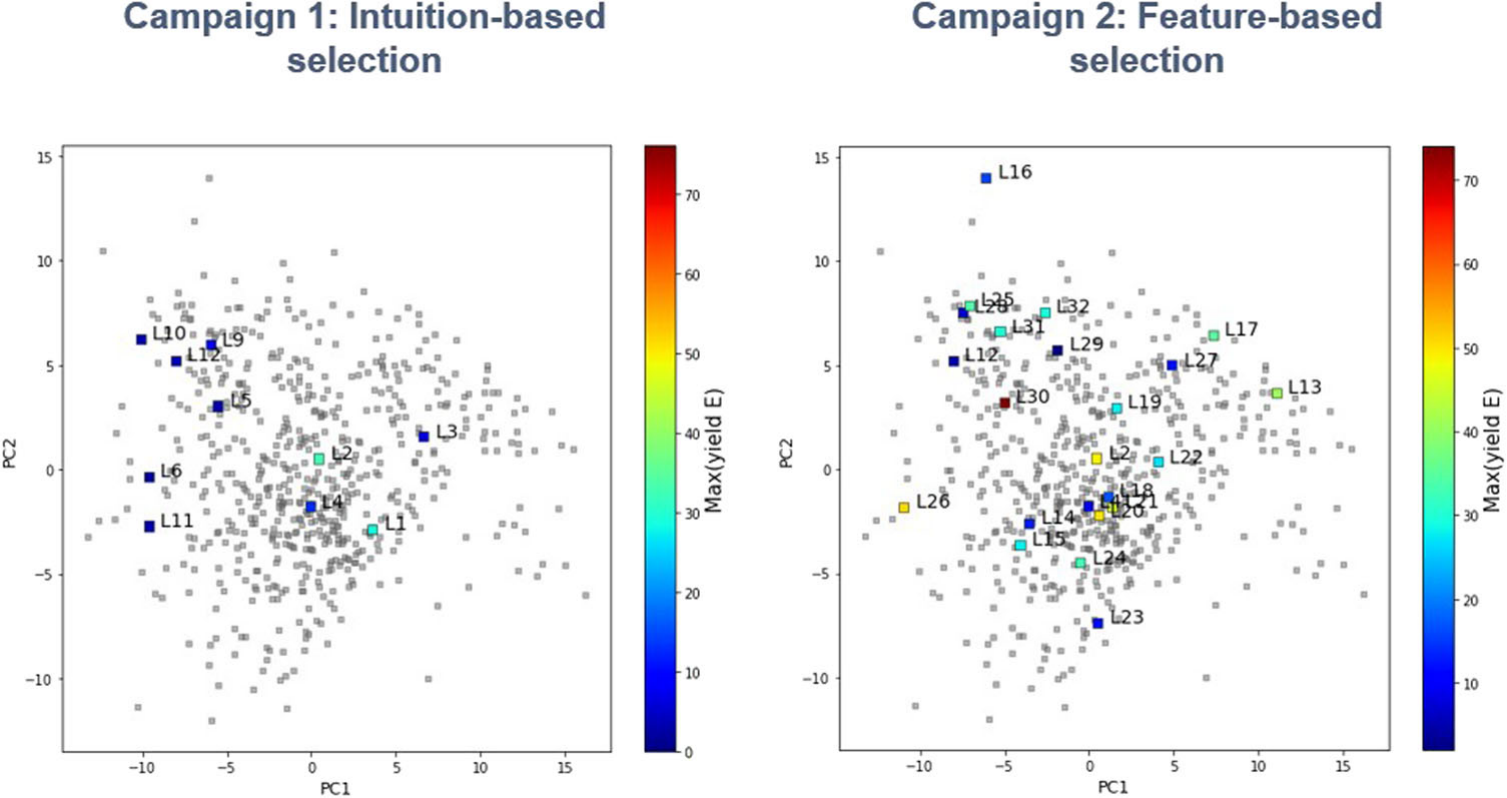
The maximum yield of 2-*E* obtained for each monodentate ligand explored in campaigns 1 and 2 mapped onto the chemical space of 365 commercial monodentate phosphines. The chemical space is represented by a two-dimensional plot of the first two principal components. Color indicates the maximum yield of 2-*E* obtained for each evaluated monodentate phosphine ligand.

Finally, comparison of the optimization results from the two campaigns superimposed on the monodentate phosphine ligand space clearly demonstrates the advantage of systematic ligand selection over chemical intuition-based ligand selection (Fig. 9). A wider range of product 2-*E* yields were observed through the systematic exploration of a diverse set of ligands, ultimately leading to the discovery of L30 as a superior ligand. This behavior can be attributed to the challenging nature of the stereoselective coupling under evaluation, where ligands typically employed in Suzuki–Miyaura couplings resulted in high conversion, yet poor *E*/*Z* selectivity. These results highlight the potential shadow that expert bias could cast on the development of creative solutions to atypical synthetic challenges, and the utility of unbiased study designs.

At this stage, the only question that remained unanswered was whether the algorithm’s optimization performance could be improved if ligands were parametrized within the algorithm instead of being treated as black-box categorical parameters. We hypothesized that the utilization of computed molecular descriptors as a means for the algorithm to relate among ligands could accelerate convergence. We therefore manually selected 15 from of the descriptors utilized in ligand selection for incorporation in a third 192-iteration optimization campaign (see Supplementary Data 1 for a list of the selected descriptors). We found that a similar optimum was reached, accessing product 2-*E* in 74% yield and 2.6:1 *E*/*Z* selectivity upon iteration 159, again under Pd-L30 catalysis. Surprisingly, the algorithm did not appear to converge as clearly as in the previous campaign because fewer iterations focused on the top ligand and, in fact, a high number of iterations focused on unproductive ligands (see Supplementary Information Table SI-12 and Fig. SI-9 for detailed results). This observation may be attributed to some form of unproductive bias introduced by the selected set of descriptors, solidifying that unbiased ligand selection was critical to the success of this optimization.

### Follow-up experiments with *E*-selective ligands identified through predictive modeling

**Fig. 10:**
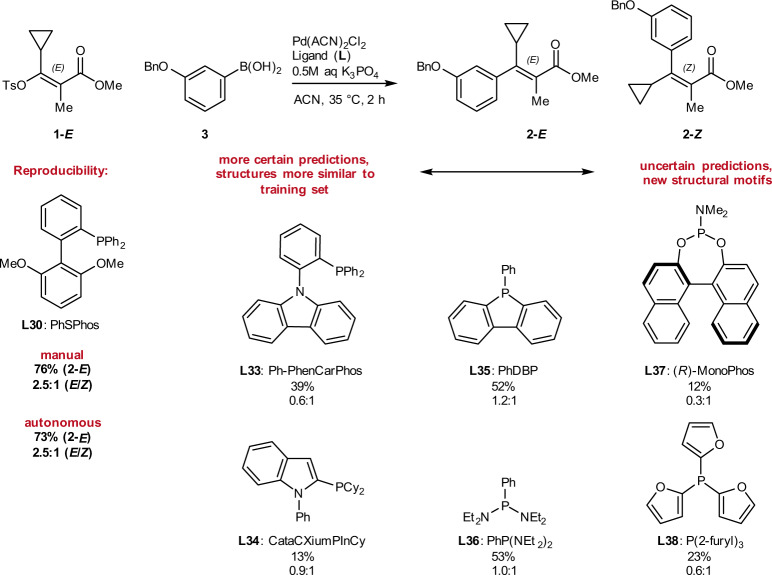
Manual experiments under optimized conditions with predicted *E*-selective ligands. Conditions: 10 µmol 1-*E*, 1 µmol 1,3,5-trimethoxybenzene, 15 µmol (3-(benzyloxy)phenyl)boronic acid 3, 0.4 µmol Pd(ACN)_2_Cl_2_, 0.9 µmol L, 30 µmol K_3_PO_4_ (0.5 M aq) in ACN (0.05 M), 2 h at 35 °C. Comparisons of the predicted and experimental yields are provided in Supplementary Information Table SI-16.

With two 192-iteration data sets in hand, various modeling strategies were employed to predict additional phosphines that could also promote *E*-selectivity^54,55^. Thus, ligands with the potential for *E*-selectivity were proposed based on a small ensemble of multivariable linear regression models, as well as proximity in chemical space. In line with the philosophy of combining exploitation and exploration, we chose six additional ligands that included structures both similar (e.g., L33, L34), and different (e.g. L36, L37) to any ligand in the training set, as well as predictions with low certainty (L38). Manual experiments employing these ligands were carried out utilizing the optimal conditions identified from autonomous optimization to measure the selectivity outcome (Fig. 10, see Supplementary Information Predictive Modeling section and Fig. SI-11 through SI-15 for details). In addition, L30 was employed in a control experiment.

Firstly, we were pleased to find that the experimental result of the manual experiment with L30 agreed very well with the results from the autonomous experimentation. Secondly, two ligands (L35, L36) out of six were identified that surpassed the 50% threshold for the yield of 2-*E*, providing a higher yield of 2-*E* than any other ligand apart from L30 in the previous experiments under the very first reaction conditions that were attempted. Further iterations of data-driven ligand suggestion, design of new ligands, and optimization of reaction conditions could possibly lead to even improved yields of 2-*E* but that was not the focus of the current study. It is important to note that the newly identified *E*-selective ligands are structurally distinct from L30 and, thus, it would have been unlikely to arrive at these selections through chemical intuition.

## Discussion

We have demonstrated the human-intervention-free multivariate optimization of a stereoselective Suzuki–Miyaura coupling in batch through the autonomous evaluation of a large phosphine ligand set and continuous parameters in tandem. This success was accomplished through a series of technological advances. First, the implementation of a robotic system capable of carrying out parallel experiments with online analytics significantly reduced cycle times. Second, the development of seamless communication between the ML algorithm and robotic hardware established a closed-loop system. Third, the incorporation of Bayesian ML algorithms, here Phoenics and Gryffin, facilitated tandem categorical and continuous parameter optimization. Finally, the employment of computed molecular features enabled the systematic and unbiased definition of the phosphine ligand search space. The application of these advances resulted in the rapid identification of optimal conditions and ligand clusters to maximize the yield of product 2-*E*.

It is relevant to ask what advantage autonomous optimization offers over more well-established process optimization strategies such as high-throughput experimentation (HTE) or design of experiments (DoE). Although we view algorithmic optimization as a complementary technology, we also believe that its advantage lies in the multivariate optimization of categorical and continuous parameters in tandem within the fewest number of iterations. To elaborate on this point, a theoretical comparison among the technologies in the context of this Suzuki–Miyaura optimization is warranted. The optimization results presented herein reveal that a distinct combination of phosphine ligand and continuous parameter selection is required for an optimal 2-*E* yield, supporting the need for a tandem categorical and continuous parameter search. A typical experimenter would be unable to thoroughly investigate a large search space involving 23 categorical parameter choices in combination with three continuous parameters within a budget of 192 experiments without some sort of statistical experimental design tool such as DoE. However, under the parameter ranges defined for this optimization, DoE studies could only hope to reveal promising reaction conditions if at least a three-level three-factor design was chosen, in order to effectively tease out trends related to phosphine to palladium ratios, which were optimal under the center points. Such a design, however, would result in a total of 621 experiments (3^3^ × 23 ligands), which is considerably higher than the 192 experimental budget to which we restricted our campaigns. Thus, both brute-force HTE and DoE would be expected to require substantially more experiments than the strategy that we have implemented.

We acknowledge that our comparisons to existing experiment planning strategies are merely theoretical, and that an experimental demonstration of the advantage of the Gryffin strategy would be preferable. However, previous baseline tests suggest the competitive advantage of our experimental planning strategy in the context of similar optimizations^32,33^. In addition, although an equal balance of explorative and exploitative sampling behaviors were implemented in this work, the precise ratio of sampling behaviors could be fine-tuned to improve future optimization performance. Finally, we acknowledge that it may not possible to rule out the possibility optimism bias in the algorithm’s predictions in the absence of a comparison between predicted values and experimental results. However, the optimism bias of a prediction is not that significant in the context of a closed-loop system, where experimental results are automatically generated and the surrogate model updated with each loop. This ability to self-correct is one of the advantages of the closed-loop system.

The autonomous optimization approach can easily be replicated to solve a multitude of multivariate process optimization problems. Once widely adopted, the technology has the potential to empower modern-day researchers to shift their focus away from routine experimental execution and toward higher-complexity problem-solving. Areas for future improvement include further extensions of algorithmic schemes to facilitate process-constrained parallel optimization as well as enhancements to multivariate data analysis to drive a better understanding of reaction trends, in essence, to learn what the algorithm learned.

## Methods

### Instrumentation

Autonomous optimization experiments were executed using a Chemspeed SWING robotic system equipped with a four-needle dispense head and four 1 ml syringe pumps to enable accurate dispenses at low volumes. Slurry dispensing was enabled through 0.8 mm needle inner diameters. Agitation was carried out through an integrated custom V&P scientific two-position tumble stirring module and temperature control was achieved through an integrated Huber Unistat chiller with temperature feedback control. Online HPLC analysis was carried out through an integrated Agilent 1100 HPLC equipped with a photodiode array detector and a custom sampling valve installed on the robot deck. See Supplementary Information General Remarks section and Fig. SI-1 for details.

### Automation of data flow

ChemOS was utilized as the scheduler that packaged proposed experiments from the ML algorithm into loops of eight for execution by the Chemspeed SWING robotic module. Communication of ChemOS with the Chemspeed SWING robot and Agilent HPLC was established through a lightweight Python tool. This script parsed experimental parameters proposed by ChemOS, calculated dispense volumes based on stock solution concentrations, and wrote those volumes to a CSV file actively being monitored by the Chemspeed AutoSuite software. Upon automated experimental execution and subsequent HPLC analysis, this script also parsed Agilent HPLC report files for peak area counts and calculated product yields based on predetermined response factors, which were then reported back to ChemOS for interpretation and proposal of new experimental parameters. See Supplementary Information Data Integration and Autonomous Optimization Protocol sections and Fig. SI-3 through SI-4 for details. See Supplementary Data 2 for an example CSV file.

### Experimental planning algorithms

The ML algorithms used for experiment planning in this study, Phoenics and Gryffin, leverage fundamental concepts from BO in combination with kernel density estimation. BO is an approach to global optimization for applications where the evaluation of a single parameter point is highly time or resource demanding. While several formulations exist, BO follows a two-step strategy to suggest parameter points for future evaluation: (1) constructing a statistical approximation to the considered experiment based on collected measurements, and (2) locating parameter points for which the approximation predicts promising performance. Phoenics and Gryffin construct the statistical approximation based on kernel density estimates of evaluated parameters and suggest promising parameter points with an explicit balance of exploitative and explorative sampling behavior with native support for parallel optimization. See Supplementary Information Machine Learning Algorithms section for details.

### Automated experimental procedure

#### General

Stock solutions or slurries were prepared manually in anhydrous ACN under N_2_ atmosphere and placed on the robot deck for autonomous execution. Two fluoropolymer and PFA mat-sealed 96-well metal blocks with 1 ml glass vial inserts were equilibrated at the designated reaction temperature under 20 psig of N_2_ with 500 rpm agitation.

#### Representative procedure for test reactions

In campaigns involving 192 iterations, eight wells from each 96-well reaction block were dedicated to standard reactions to test for reproducibility. To each well was dispensed Pd(ACN)_2_Cl_2_ (0.25 µmol, 25 µl of 0.01 M stock solution) and L2 (PoTol_3_) (0.38 µmol, 19 µl of 0.02 M stock mixture), followed by 7 min of age time. Then, *E*-tosylate 1-*E* (10 µmol) with 1,3,5-trimethoxybenzene (1 µmol) was dispensed (20 µl of 0.5 M/0.05 M stock solution), followed by (3-(benzyloxy)phenyl)boronic acid 3 (15 µmol, 30 µl of 0.5 M stock solution in degassed ACN 5% H_2_O), followed by anhydrous ACN (106 µl) to ensure a total organic solvent volume of 200 µl. Then, a dispense of degassed aqueous K_3_PO_4_ (30 µmol, 60 µl of 0.5 M stock solution) was carried out to initiate the reaction. This procedure was executed sequentially for each well within a loop of eight replicates in 15-min intervals. Each replicate was time stamped individually, aged for 120 min, and sampled for online analysis.

#### Representative procedure for optimization reactions

In campaigns involving 192 iterations, 88 wells from each 96-well reaction block were dedicated to the optimization reactions. To each well was dispensed Pd(ACN)_2_Cl_2_ (0.1–0.5 µmol, 10–50 µl of 0.01 M stock solution) and phosphine ligand (0.05–2.0 µmol, 3–100 µl of 0.02 M stock mixture), followed by 7 min of age time. Then, E-tosylate 1-*E* (10 µmol) with 1,3,5-trimethoxybenzene (1.0 µmol) was dispensed (20 µl of 0.5 M/ 0.05 M stock solution), followed by (3-(benzyloxy)phenyl)boronic acid 3 (15 µmol, 30 µl of 0.5 M stock solution in degassed ACN 5% H_2_O), followed by anhydrous ACN (0–138 µl) to ensure a total organic solvent volume of 200 µl. Then, a dispense of degassed aqueous K_3_PO_4_ (30 µmol, 60 µl of 0.5 M stock solution) was carried out to initiate the reaction. This procedure was executed sequentially for each well within a loop of eight experiments in 15-min intervals. Each reaction was time stamped individually, aged for 120 min, and sampled for online analysis.

#### Sampling and analysis

Two polypropylene 96-well collection blocks sealed with a silicone mats were manually prefilled with 800 µl of acetonitrile 10% aqueous pH 3.5 ammonium formate buffer and placed on the robot deck. Upon reaching the reaction endpoint at 120 min, 10 µl of reaction mixture was aliquoted and dispensed into the 800 µl quench solution in the collection block. Upon needle-mixing, 40 µl of quenched sample from the collection block was aliquoted and injected to the on-deck sampling valve outfitted with a 5 µl loop. The valve was automatically switched to transfer the sample to the Agilent 1100 HPLC for analysis.

See Supplementary Information Supplementary Methods section as well as Table SI-1 through SI-5 and Fig. SI-2 for details. See Supplementary Movie 1 for a recording of the robot deck during experimental execution.

## Supplementary information

Supplementary Information

Description of Additional Supplementary Files

Supplementary Movie 1

Supplementary Movie 2

Supplementary Data 1

Supplementary Data 2

## Data Availability

All data generated during this study are included in this published article, the Supplementary Information Analytical Data section, Supplementary Information Table SI-6 through SI-13, Supplementary Information Fig. SI-5 through SI-10, Supplementary Data 1 through 2, and Supplementary Movie 1 through 2. The computed molecular features utilized in this study have been made publicly available at https://kraken.cs.toronto.edu/dashboard^45^.

## Code availability

The custom code used in this study has been made publicly available on GitLab at https://gitlab.com/heingroup/cs-auto-optimization under the MIT open source license.

## References

[1] Jensen, K. F., Coley, C. W. & Eyke, N. S. Autonomous discovery in the chemical sciences part I: progress. *Angew. Chem. Int. Ed*. **59**, 22858–22893 (2019). 10.1002/anie.201909987 31553511

[2] Coley, C. W., Eyke, N. S. & Jensen, K. F. Autonomous discovery in the chemical sciences part II: outlook. *Angew. Chem. Int. Ed*. **59**, 23414–23436 (2019). 10.1002/anie.201909989 31553509

[3] Häse F, Roch LM, Aspuru-Guzik A (2019). Next-generation experimentation with self-driving laboratories. Trends Chem..

[4] Stein HS, Gregoire JM (2019). Progress and prospects for accelerating materials science with automated and autonomous workflows. Chem. Sci..

[5] Desai B (2013). Rapid discovery of a novel series of Abl kinase inhibitors by application of an integrated microfluidic synthesis and screening platform. J. Med. Chem..

[6] Weber L, Wallbaum S, Broger C, Gubernator K (1995). Optimization of the biological activity of combinatorial compound libraries by a genetic algorithm. Angew. Chem. Int. Ed..

[7] Porwol, L. et al. An autonomous chemical robot discovers the rules of inorganic coordination chemistry without prior knowledge. *Angew. Chem. Int. Ed*. **59**, 11256–11261 (2020). 10.1002/anie.202000329 PMC7384156 32419277

[8] Tabor DP (2018). Accelerating the discovery of materials for clean energy in the era of smart automation. Nat. Rev. Mater..

[9] Nikolaev P (2016). Autonomy in materials research: a case study in carbon nanotube growth. npj Comput. Mater..

[10] Granda JM, Donina L, Dragone V, Long DL, Cronin L (2018). Controlling an organic synthesis robot with machine learning to search for new reactivity. Nature.

[11] Sans V, Cronin L (2016). Towards dial-a-molecule by integrating continuous flow, analytics and self-optimisation. Chem. Soc. Rev..

[12] Sans V, Porwol L, Dragone V, Cronin L (2015). A self optimizing synthetic organic reactor system using real-time in-line NMR spectroscopy. Chem. Sci..

[13] Vasudevan, N. et al. Direct C‐H arylation of indole‐3‐acetic acid derivatives enabled by an autonomous self‐optimizing flow reactor. *Adv. Synth. Catal*. **363**, 791–799 (2020).

[14] Mateos C, Nieves-Remacha MJ, Rincón JA (2019). Automated platforms for reaction self-optimization in flow. React. Chem. Eng..

[15] Clayton AD (2019). Algorithms for the self-optimisation of chemical reactions. React. Chem. Eng..

[16] Bédard A-C (2018). Reconfigurable system for automated optimization of diverse chemical reactions. Science.

[17] Cortés-Borda D (2018). An autonomous self-optimizing flow reactor for the synthesis of natural product carpanone. J. Org. Chem..

[18] Hsieh HW, Coley CW, Baumgartner LM, Jensen KF, Robinson RI (2018). Photoredox iridium-nickel dual-catalyzed decarboxylative arylation cross-coupling: from batch to continuous flow via self-optimizing segmented flow reactor. Org. Process Res. Dev..

[19] Zhou Z, Li X, Zare RN (2017). Optimizing chemical reactions with deep reinforcement learning. ACS Cent. Sci..

[20] Reizman BJ, Jensen KF (2016). Feedback in flow for accelerated reaction development. Acc. Chem. Res..

[21] Fitzpatrick DE, Battilocchio C, Ley SV (2016). A novel internet-based reaction monitoring, control and autonomous self-optimization platform for chemical synthesis. Org. Process Res. Dev..

[22] Cortés-Borda D (2016). Optimizing the Heck−Matsuda reaction in flow with a constraint-adapted direct search algorithm. Org. Process Res. Dev..

[23] McMullen JP, Jensen KF (2010). An automated microfluidic system for online optimization in chemical synthesis. Org. Process Res. Dev..

[24] Baumgartner LM, Coley CW, Reizman BJ, Gao KW, Jensen KF (2018). Optimum catalyst selection over continuous and categorical process variables with a single droplet microfluidic reaction platform. React. Chem. Eng..

[25] Reizman BJ, Wang Y-M, Buchwald SL, Jensen KF (2016). Suzuki-Miyaura cross-coupling optimization enabled by automated feedback. React. Chem. Eng..

[26] Reizman BJ, Jensen KF (2015). Simultaneous solvent screening and reaction optimization in microliter slugs. Chem. Commun..

[27] Burger B (2020). A mobile robotic chemist. Nature.

[28] MacLeod BP (2020). Self-driving laboratory for accelerated discovery of thin-film materials. Sci. Adv..

[29] Gongora AE (2020). A Bayesian experimental autonomous researcher for mechanical design. Sci. Adv..

[30] Langner S (2020). Beyond ternary OPV: high‐throughput experimentation and self‐driving laboratories optimize multicomponent systems. Adv. Mater..

[31] Frazier, P. I. A Tutorial on Bayesian optimization. Preprint at https://arxiv.org/abs/1807.02811 (2018).

[32] Häse F, Roch LM, Kreisbeck C, Aspuru-Guzik A (2018). Phoenics: a Bayesian optimizer for chemistry. ACS Cent. Sci..

[33] Häse, F., Aldeghi, M., Hickman, R. J., Roch, L. M., & Aspuru-Guzik, A. Gryffin: An algorithm for Bayesian optimization of categorical variables informed by expert knowledge. *Appl. Phys. Rev.* **8**, 031406 (2021).

[34] Plummer CW (2017). Design and synthesis of novel, selective GPR40 AgoPAMs. ACS Med. Chem. Lett..

[35] Christensen M (2016). Enantioselective synthesis of α-methyl-β-cyclopropyldihydrocinnamates. J. Org. Chem..

[36] Clayden, J., Greeves, N., & Warren, S. G. *Organic Chemistry* (Oxford Univ. Press, 2012).

[37] Chehal NK, Budzelaar PHM, Hultin PG (2018). E - Z isomerization in Suzuki cross-couplings of haloenones: Ligand effects and evidence for a separate catalytic cycle. Org. Biomol. Chem..

[38] Li BX (2017). Highly stereoselective synthesis of tetrasubstituted acyclic all-carbon olefins via enol tosylation and Suzuki-MIyaura coupling. J. Am. Chem. Soc..

[39] Molinaro C (2015). Catalytic, asymmetric, and stereodivergent synthesis of non-symmetric β,β-Diaryl-α-Amino Acids. J. Am. Chem. Soc..

[40] Lu GP, Voigtritter KR, Cai C, Lipshutz BH (2012). Ligand effects on the stereochemical outcome of Suzuki-Miyaura couplings. J. Org. Chem..

[41] Johansson Seechurn CCC, Kitching MO, Colacot TJ, Snieckus V (2012). Palladium-catalyzed cross-coupling: a historical contextual perspective to the 2010 nobel prize. Angew. Chem. Int. Ed..

[42] Martin R, Buchwald SL (2008). Palladium-catalyzed Suzuki-Miyaura cross-coupling reactions employing dialkylbiaryl phosphine ligands. Acc. Chem. Res..

[43] Nguyen HN, Huang X, Buchwald SL (2003). The first general palladium catalyst for the Suzuki-Miyaura and carbonyl enolate coupling of aryl arenesulfonates. J. Am. Chem. Soc..

[44] Littke AF, Dai C, Fu GC (2000). Versatile catalysts for the Suzuki cross-coupling of arylboronic acids with aryl and vinyl halides and triflates under mild conditions. J. Am. Chem. Soc..

[45] Gensch, T. et al. A comprehensive discovery platform for organophosphorus ligands for catalysis. Preprint at 10.26434/chemrxiv.12996665.v1 (2021). 10.1021/jacs.1c09718 35020383

[46] Roch LM (2020). ChemOS: an orchestration software to democratize autonomous discovery. PLoS ONE.

[47] Christensen M (2019). Development of an automated kinetic profiling system with online HPLC for reaction optimization. React. Chem. Eng..

[48] Häse F, Roch LM, Aspuru-Guzik A (2018). Chimera: enabling hierarchy based multi-objective optimization for self-driving laboratories. Chem. Sci..

[49] Vellanki, P. et al. Process-constrained batch Bayesian optimisation. Preprint at https://papers.nips.cc/paper/2017/file/1f71e393b3809197ed66df836fe833e5-Paper.pdf (2017).

[50] Durand DJ, Fey N (2019). Computational ligand descriptors for catalyst design. Chem. Rev..

[51] Santiago CB, Guo J-Y, Sigman MS (2018). Predictive and mechanistic multivariate linear regression models for reaction development. Chem. Sci..

[52] Wu K, Doyle AG (2017). Parameterization of phosphine ligands demonstrates enhancement of nickel catalysis via remote steric effects. Nat. Chem..

[53] Niemeyer ZL, Milo A, Hickey DP, Sigman MS (2016). Parameterization of phosphine ligands reveals mechanistic pathways and predicts reaction outcomes. Nat. Chem..

[54] Gensch, et al. Design and application of a training set for monophosphine ligands in metal catalysis. Preprint at 10.26434/chemrxiv.13160939 (2021).

[55] Zhao S (2018). Enantiodivergent Pd-catalyzed C–C bond formation enabled through ligand parameterization. Science.

