# Supplementary material for: Data-science driven autonomous process optimization
Source: Commun Chem. 2021 Aug 2;4:112. doi: 10.1038/s42004-021-00550-x (PMC9814253; doi:10.1038/s42004-021-00550-x)
Supplement: Supplementary file 1 — Supplementary Information [file 42004_2021_550_MOESM1_ESM.pdf]

SUPPLEMENTARY INFORMATION

DATA-SCIENCE DRIVEN AUTONOMOUS PROCESS OPTIMIZATION

Melodie Christensen[1,2], Lars P. E. Yunker[1], Folarin Adedeji[2], Florian Häse[3,4,5,6,7], Loïc M. Roch[3,4,5,7], Tobias Gensch[8], Gabriel dos Passos Gomes[4,5,6], Tara Zepel[1], Matthew S. Sigman*[8], Alán Aspuru-Guzik*[3,4,5,6,9] and Jason E. Hein[1*]

[1]Department of Chemistry, University of British Columbia, Vancouver, British Columbia V6T 1Z1, Canada.
[2]Department of Process Research and Development, Merck & Co., Inc., Rahway, NJ 07065, United States.
[3]Department of Chemistry and Chemical Biology, Harvard University, Cambridge, MA 02138, United States.
[4]Department of Chemistry, University of Toronto, Toronto, Ontario M5S 3H6, Canada.
[5]Department of Computer Science, University of Toronto, Toronto, Ontario M5T 3A1, Canada.
[6]Vector Institute for Artificial Intelligence, Toronto, Ontario M5S 1M1, Canada.
[7]ChemOS Sàrl, Lausanne, Vaud 1006, Switzerland.
[8]Department of Chemistry, University of Utah, Salt Lake City, Utah 84112, United States.
[9]Canadian Institute for Advanced Research, Toronto, Ontario M5G 1Z8, Canada.

**Corresponding authors: Matthew S. Sigman sigman@chem.utah.edu, Alán Aspuru-Guzik aspuru@utoronto.ca, Jason E. Hein jhein@chem.ubc.ca*

Table of Contents

DATA-SCIENCE DRIVEN AUTONOMOUS PROCESS OPTIMIZATION 1

1. General remarks 3

1.1 Chemical suppliers 3

1.2 Equipment 3

1.3 Analytical methods 4

2. Supplementary Methods 5

2.1 Procedure for stock mixture preparations 5

2.2 Procedure for autonomous optimization experiments 9

2.3 Procedure for manual experiments 10

2.4 Procedures for the preparation of non-commercial compounds 11

3. Data Integration 13

4. Autonomous optimization protocol 14

4.1 Csv file with dispense volumes 14

4.2 Sample Chemspeed Autosuite protocol 14

5. Machine learning algorithms 16

5.1 Process constrained optimization in the context of autonomous experimentation 17

6. Analytical data 18

6.1 ^{1}H NMR weight % purity determinations 18

6.2 Calibration curves 20

6.3 Preliminary data 21

6.4 Campaign 1 data 22

6.5 Campaign 2 data 25

6.6 Campaign 3 data 29

6.7 Campaign 3 visualization 34

6.8 Standard experiments 35

7. Descriptor computation 36

8. Training set selection 37

9. Predictive modeling 38

9.1 Regression modeling 38

9.2 Classification suggestion 40

9.3 Prediction results 41

1. General remarks

1.1 Chemical suppliers

Commercial reagents were purchased from Millipore Sigma, Oakwood, Combi-Blocks, Alfa Aesar, Acros, Strem, Apollo and Alichem; and used as received. Anhydrous solvents were purchased from Millipore Sigma and used as received. Aqueous solutions were deoxygenated through subsurface nitrogen sparge for a minimum of 30 minutes.

1.2 Equipment

Flash chromatography was performed on a CombiFlash purification system employing Redisep Rf Gold silica gel columns. ^{1}H NMR spectra for weight % purity determinations were recorded on a Bruker 500 MHz instrument with a delay time of 20 sec in 32 scans. Samples were prepared in $CDCl_3$ and spectra were calibrated to the $CDCl_3$ reference peak at 7.26 ppm.

Autonomous optimization experiments were carried out on a Chemspeed Swing liquid handling robot equipped with an adjustable-pitch four needle (0.8 mm ID) liquid dispense tool, a V&P Scientific tumble stirrer and a Huber Unistat 82T chiller. The robot was integrated with an Agilent 1100 HPLC-UV system through installation of a two position, 6-port HPLC valve on the Chemspeed Swing deck and connection to the Agilent 1100 through 0.17 mm ID tubing. Valve switching and chromatographic resolutions were triggered through contact closures. A 5 µL sample loop was installed.

Reactions were carried out in 96-well aluminum reaction blocks (Analytical Sales 96973) with 8 x 30 mm glass vial inserts (Analytical Sales 884001). Vials were sealed with a layer of two PFA films (Analytical Sales 96967), one tan fluoropolymer mat (Analytical Sales 78040) and a custom aluminum needle-guiding top cover (figure SI-1). Vigorous agitation was achieved with 5 mm PVDF-encapsulated NdFeB magnetic tumble stir discs (VP 772DP-N42-5-2). Reaction aliquots were sampled into 96-well dilution plates (Analytical Sales 17P687) capped with pre-slit silicone/PTFE cap mats (Analytical Sales 965075).

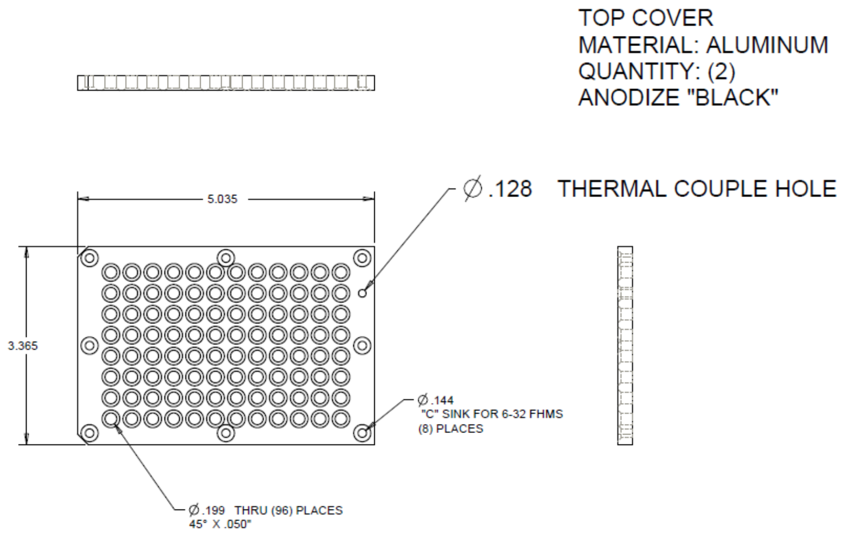

Figure SI-1. Custom aluminum needle-guiding top cover.

1.3 Analytical methods

Reaction analysis was carried out through utilization of the HPLC-UV method described in Table SI-1.

Table SI-1. HPLC-UV method for yield determination

Column:	Cortecs C18 2.7 µm, 4.6 x 150 mm	
Column Temperature:	55 °C	
Flow Rate:	1.5 ml/min	
Detection:	210 nm	
Acquisition Time:	10 min	
Mobile Phase:	Solvent A = 2 mM ammonium formate in water; Solvent B = 2 mM ammonium formate in acetonitrile 10% water	
Mobile Phase Program:	Time<br>0.00 min<br>6.00 min<br>8.00 min<br>8.10 min<br>10.00 min	B%<br>5<br>95<br>95<br>5<br>5
Injection Volume:	5 µL	
Compound Name:	Retention time:	
3-(benzyloxy)phenyl)boronic acid (**3**)	4.75 min	
1,3,5-trimethoxybenzene	4.93 min	
(*E*)-Methyl 3-cyclopropyl-2-methyl-3-(tosyloxy)acrylate (**1-*E***)	6.13 min	
(*Z*)-Methyl 3-(3-(benzyloxy)phenyl)-3-cyclopropyl-2-methylacrylate (**2-*Z***)	6.98 min	
(*E*)-Methyl 3-(3-(benzyloxy)phenyl)-3-cyclopropyl-2-methylacrylate (**2-*E***)	7.10 min	
Example Chromatogram		

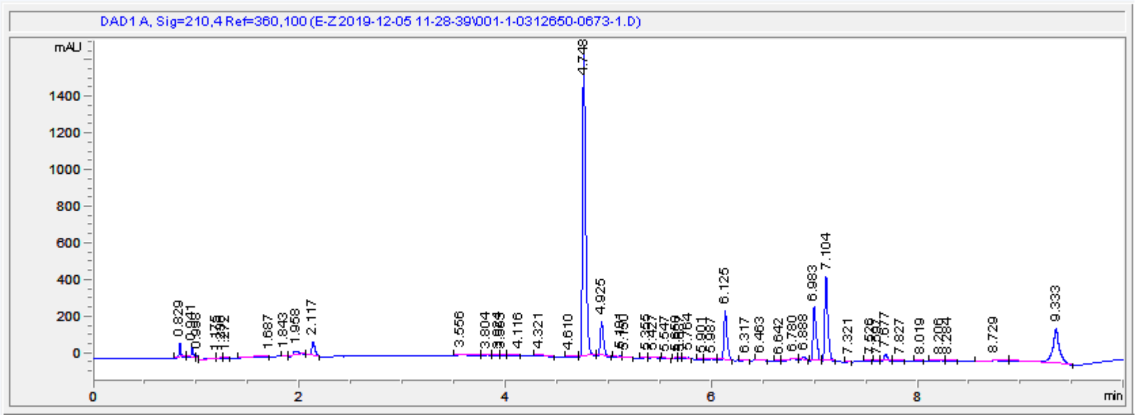

2. Supplementary Methods

2.1 Procedure for stock mixture preparations

Fresh stock mixtures were prepared in 4 - 20 ml vials in a positive nitrogen pressure glove box, sealed with septa caps and transferred to the positive nitrogen pressure Chemspeed robot deck. Stock mixture concentrations were selected based on factors such as solubility, minimum accurate dispense volume, and a 200 µl total organic reaction volume (Tables SI-2 and SI-3). The mixtures were prepared by weighing the solids or oils on an analytical balance followed by adding anhydrous ACN through manual pipetting. The recipes followed in stock mixture preparation are provided in Tables SI-4 and SI-5.

Table SI-2. Campaign 1 stock mixture concentrations and parameter ranges

**Chemical**	**Eq low**	**Eq high**	**µmol low**	**µmol high**	**µl low**	**µl high**	**M**
$Pd(ACN)_2Cl_2$	0.01	0.05	0.1	0.5	10	50	0.010
P Ligand	0.005	0.20	0.05	2	1	50	0.040
E-Tosyl (**1-*E***)	1.00	1.00	10	10	20	20	0.500
Internal Std*	0.10	0.10	1	1			0.050
ArBA (**3**)	1.00	2.00	10	20	40	80	0.250
K_3PO_4	3.00	3.00	30	30	60	60	0.500
ACN					129		
Total Org					200	200	
Total Aq					60	60	

*1,3,5-trimethoxybenzene

Table SI-3. Campaigns 2 and 3 stock mixture concentrations and parameter ranges

**Chem**	**Eq low**	**Eq high**	**µmol low**	**µmol high**	**µl low**	**µl high**	**M**
$Pd(ACN)_2Cl_2$	0.01	0.05	0.1	0.5	10	50	0.010
P Ligand	0.01	0.20	0.05	2	3	100	0.020
E-Tosyl (**1-*E***)	1.00	1.00	10	10	20	20	0.500
Internal Std*	0.10	0.10	1	1			0.050
ArBA (**3**)	1.50	1.50	15	15	30	30	0.500
K_3PO_4	3.00	3.00	30	30	60	60	0.500
ACN					138		
Total Org					200	200	
Total Aq					60	60	

*1,3,5-trimethoxybenzene

Table SI-4. Campaign 1 stock mixture recipes

**Mixture**	**Chemical**	**MW (g/mol)**	**Amount (mg)**	**Density (g/ml)***	**Amount (ml)**
$Pd(ACN)_2Cl_2$	14592-56-4	259.43	31.13	1.00	0.03
	ACN	41.05		0.79	11.97
PPh_3 (**L1**)	603-35-0	262.29	125.90	1.00	0.13
	ACN	41.05		0.79	11.87
$PoTol_3$ (**L2**)	6163-58-2	304.37	146.10	1.00	0.15
	ACN	41.05		0.79	11.85
$PtBu_3.HBF_4$ (**L3**)	131274-22-1	202.32	97.11	1.00	0.10
	ACN	41.05		0.79	11.90
AtaPhos (**L4**)	932710-63-9	265.38	127.38	1.00	0.13
	ACN	41.05		0.79	11.87
MorDalPhos (**L5**)	1237588-12-3	463.65	222.55	1.00	0.22
	ACN	41.05		0.79	11.78
QPhos (**L6**)	312959-24-3	710.72	341.15	1.00	0.34
	ACN	41.05		0.79	11.66
DPPF (**L7**)	12150-46-8	554.39	266.11	1.00	0.27
	ACN	41.05		0.79	11.73
DTBPF (**L8**)	84680-95-5	474.43	227.73	1.00	0.23
	ACN	41.05		0.79	11.77
XPhos (**L9**)	564483-18-7	476.73	228.83	1.00	0.23
	ACN	41.05		0.79	11.77
BrettPhos (**L10**)	1070663-78-3	536.78	257.65	1.00	0.26
	ACN	41.05		0.79	11.74
AdBrettPhos (**L11**)	1160861-59-5	640.93	307.65	1.00	0.31
	ACN	41.05		0.79	11.69
RockPhos (**L12**)	1262046-34-3	468.71	224.98	1.00	0.22

	ACN	41.05		0.79	11.78
E-Tos	E-Tos (**1-*E***)	310.36	744.87	1.00	0.74
	1,3,5-trimethoxybenzene	168.19	40.37	1.00	0.04
	ACN	41.05		0.79	4.01
ArBA	ArBA (**3**)	228.05	1094.64	1.00	1.09
	Water	18.02		1.00	0.91
	ACN	41.05		0.79	18.11
K_3PO_4	potassium phosphate, tribasic	212.27	3056.62	1.00	3.06
	ACN	41.05		0.79	25.74

*Density of 1.00 g/ml was assumed for all solids.

Table SI-5. Campaigns 2 and 3 stock mixture recipes

**Mixture**	**Chemical**	**MW**	**Amount (mg)**	**Density (g/ml)***	**Amount (ml)**
$Pd(ACN)_2Cl_2$	14592-56-4	259.43	37.36	1.00	0.04
	ACN	41.05		0.79	14.36
PEt_3 (**L13**)	554-70-1	118.16	17.02	0.80	0.0212
	ACN	41.05		0.79	7.18
PCy_2Ph (**L19**)	6476-37-5	274.38	39.51	1.00	0.04
	ACN	41.05		0.79	7.16
1-Cy_2P-2’,4’,6’-MeO-Ph_2 (**L28**)	1000171-05-0	440.55	63.44	1.00	0.06
	ACN	41.05		0.79	7.14
PPh_2(2,6-Me-Ph) (**L20**)	117672-33-0	290.34	41.81	1.00	0.04
	ACN	41.05		0.79	7.16
PPh_2(2-OMe-Ph) (**L21**)	53111-20-9	292.31	42.09	1.00	0.04
	ACN	41.05		0.79	7.16
PallylPh_2 (**L22**)	2741-38-0	226.25	32.58	1.05	0.03
	ACN	41.05		0.79	7.17
JohnPhos (**L29**)	224311-51-7	298.40	42.97	1.00	0.04
	ACN	41.05		0.79	7.16

$P(C_6F_5)_2Ph$ (**L23**)	5074-71-5	442.20	63.68	1.00	0.06
	ACN	41.05		0.79	7.14
RockPhos (**L12**)	1262046-34-3	468.69	67.49	1.00	0.07
	ACN	41.05		0.79	7.13
PhAtaPhos (**L24**)	739-58-2	305.35	43.97	1.00	0.04
	ACN	41.05		0.79	7.16
SPhos (**L31**)	657408-07-6	410.53	59.12	1.00	0.06
	ACN	41.05		0.79	7.14
(R)SITCP (**L18**)	856407-37-9	354.42	51.04	1.00	0.05
	ACN	41.05		0.79	7.15
P(mesityl)$_3$ (**L14**)	23897-15-6	388.52	55.95	1.00	0.06
	ACN	41.05		0.79	7.14
P(3,5-Me-4-OMe-Ph)$_3$ (**L15**)	121898-64-4	436.52	62.86	1.00	0.06
	ACN	41.05		0.79	7.14
Et-PhenCarPhos (**L32**)	1308652-66-5	331.39	47.72	1.00	0.05
	ACN	41.05		0.79	7.15
PoTol$_3$ (**L2**)	6163-58-2	304.37	43.83	1.00	0.04
	ACN	41.05		0.79	7.16
$Ph_2P(CH_2)_3Si(OEt)_3$ (**L25**)	52090-23-0	390.53	56.24	1.00	0.06
	ACN	41.05		0.79	7.14
PPh(4-(2,2-CF$_3$-F$_7$pent)-Ph)$_2$ (**L26**)	322647-83-6	926.41	133.40	1.00	0.13
	ACN	41.05		0.79	7.07
m-CroPhos (**L27**)	1620882-90-7	200.30	28.84	1.00	0.03
	ACN	41.05		0.79	7.17
AtaPhos (**L4**)	932710-63-9	265.37	38.21	1.00	0.04
	ACN	41.05		0.79	7.16
PhSPhos (**L30**)	819867-24-8	398.43	57.37	1.00	0.06
	ACN	41.05		0.79	7.14

$P(nOct)_3$ (**L16**)	4731-53-7	370.64	53.37	0.83	0.06
	ACN	41.05		0.79	7.14
$PnPr_3$ (**L17**)	2234-97-1	160.24	23.07	0.80	0.0288
	ACN	41.05		0.79	
E-Tos	E-Tos (**1-*E***)	310.36	893.85	1.00	0.89
	1,3,5-trimethoxybenzene	168.19	48.44	1.00	0.05
	ACN	41.05		0.79	4.82
ArBA	ArBA (**3**)	228.05	985.18	1.00	0.99
	Water	18.02		1.00	0.38
	ACN	41.05		0.79	7.65
K_3PO_4	potassium phosphate, tribasic	212.27	2445.29	1.00	2.45
	ACN	41.05		0.79	20.59

*Density of 1.00 g/ml was assumed for all solids.

2.2 Procedure for autonomous optimization experiments

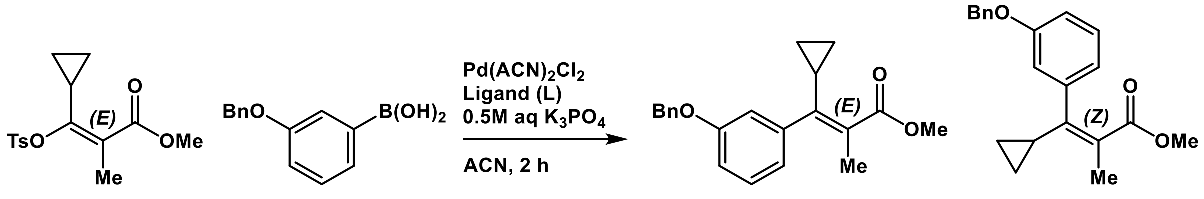

1-*E* **3** **2-*E*** **2-*Z***

General: Two fluoropolymer and PFA mat-sealed 96-well metal blocks with 1 mL glass vial inserts were equilibrated at the designated reaction temperature under 20 psig of nitrogen with 500 rpm agitation.

Representative procedure for test reactions: In campaigns involving 192 iterations, eight wells from each 96-well reaction block were dedicated to standard reactions to test for reproducibility. To each well was dispensed $Pd(ACN)_2Cl_2$ (0.25 µmol, 25 µl of 0.01 M stock solution), and **L2** ($PoTol_3$) (0.38 µmol, 19 µl of 0.02 M stock mixture), followed by 7 min of age time. Then, *E*-tosylate **1-*E*** (10 µmol) with 1,3,5-trimethoxybenzene (1 µmol) was dispensed (20 µl of 0.5 M/ 0.05 M stock solution), followed by (3-(benzyloxy)phenyl)boronic acid **3** (15 µmol, 30 µl of 0.5 M stock solution in degassed ACN 5% H_2O), followed by anhydrous ACN (106 µl) to ensure a total organic solvent volume of 200 µl. Then, a dispense of degassed aqueous K_3PO_4 (30 µmol, 60 µl of 0.5 M stock solution) was carried out to initiate the reaction. This procedure was executed sequentially for each well within a loop of eight replicates in 15-minute intervals. Each replicate was time stamped individually, aged for 120 min, and sampled for online analysis.

Representative procedure for optimization reactions: In campaigns involving 192 iterations, 88 wells from each 96-well reaction block were dedicated to the optimization reactions. To each well was dispensed $Pd(ACN)_2Cl_2$ (0.1 - 0.5 µmol, 10 - 50 µl of 0.01 M stock solution), and phosphine ligand (0.05 - 2.0 µmol, 3 - 100 µl of 0.02 M stock mixture), followed by 7 min of age time. Then, E-tosylate **1-*E*** (10 µmol) with 1,3,5-

trimethoxybenzene (1.0 µmol) was dispensed (20 µl of 0.5 M/ 0.05 M stock solution), followed by (3-(benzyloxy)phenyl)boronic acid **3** (15 µmol, 30 µl of 0.5 M stock solution in degassed ACN 5% H_2O), followed by anhydrous ACN (0 - 138 µl) to ensure a total organic solvent volume of 200 µl. Then, a dispense of degassed aqueous K_3PO_4 (30 µmol, 60 µl of 0.5 M stock solution) was carried out to initiate the reaction. This procedure was executed sequentially for each well within a loop of eight experiments in 15-minute intervals. Each reaction was time stamped individually, aged for 120 min, and sampled for online analysis.

Sampling and analysis: Two polypropylene 96-well collection blocks sealed with a silicone mats were manually prefilled with 800 µl of acetonitrile 10% aqueous pH 3.5 ammonium formate buffer and placed on the robot deck. Upon reaching the reaction end point at 120 min, 10 ul of reaction mixture was aliquoted and dispensed into the 800 ul quench solution in the collection block. Upon needle-mixing, 40 ul of quenched sample from the collection block was aliquoted and injected to the on-deck sampling valve outfitted with a 5 µl loop. The valve was automatically switched to transfer the sample to the Agilent 1100 HPLC for analysis.

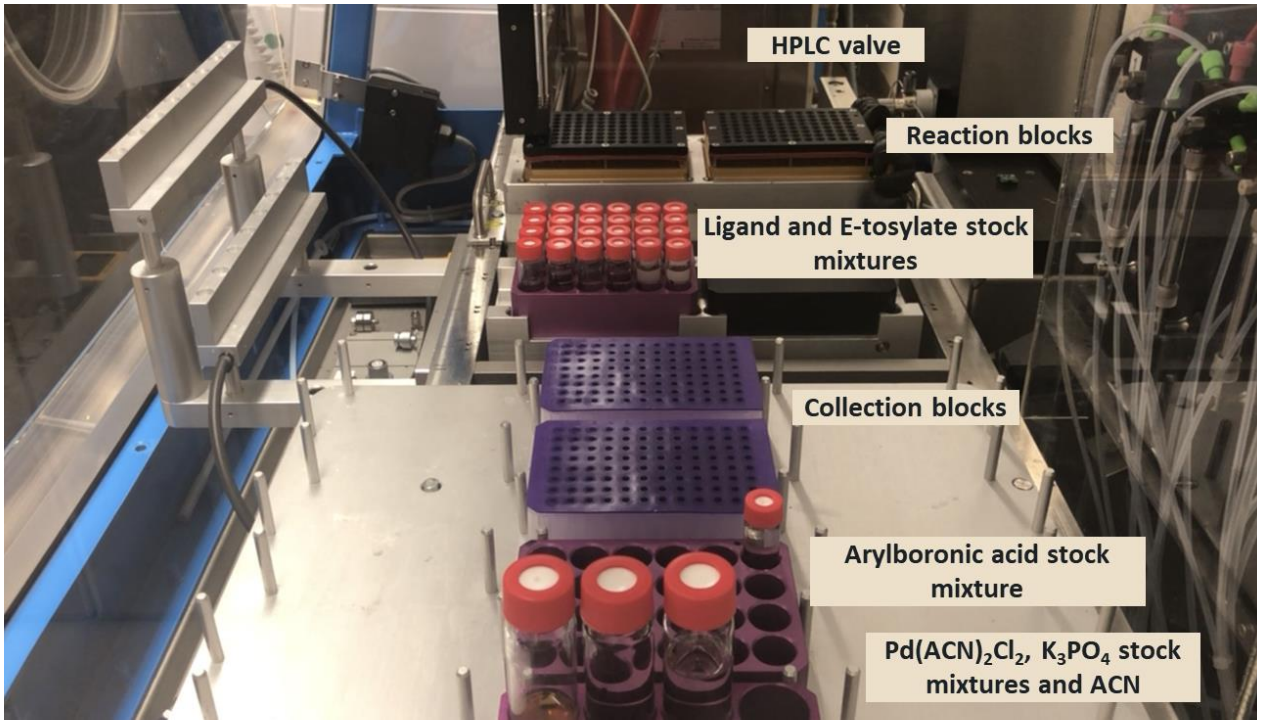

Figure SI-2: Chemspeed robot deck during autonomous experiments.

2.3 Procedure for manual experiments

Manual experiments were carried out in in a positive nitrogen pressure glove box, in 96-well aluminum reaction blocks with 8 x 30 mm glass vial inserts. To each well was pipet dispensed $Pd(ACN)_2Cl_2$ (0.38 µmol, 38 µl of 0.01 M stock solution), and phosphine ligand (0.92 µmol, 46 µl of 0.02 M stock mixture), followed by 7 min of age time. Then, *E*-tosylate **1-*E*** (10 µmol) with 1,3,5-trimethoxybenzene (1 µmol) was pipet dispensed (20 µl of 0.5 M/ 0.05 M stock solution), followed by (3-(benzyloxy)phenyl)boronic acid **3** (15 µmol, 30 µl of 0.5 M stock solution in degassed ACN 5% H_2O), followed by anhydrous ACN (66 µl) to ensure a total organic solvent volume of 200 µl. Then, degassed aqueous K_3PO_4 (30 µmol, 60 µl of 0.5 M stock solution) was pipet dispensed. The block was sealed and agitated for 120 minutes at 35 °C under 500 rpm

tumble stirring. Upon reaction completion, 10 ul of reaction mixture was aliquoted and quenched into 800 µl of acetonitrile 10% aqueous pH 3.5 ammonium formate buffer. Each sample was analyzed via the Chemspeed online HPLC for consistency.

2.4 Procedures for the preparation of non-commercial compounds

*Methyl 3-cyclopropyl-2-methyl-3-oxopropanoate **5**:*

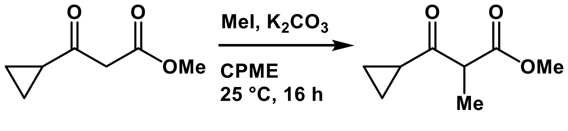

4 **5**

To a round-bottomed flask under nitrogen atmosphere was charged methyl 3-cyclopropyl-3-oxopropanoate **4** (40.0 g, 281 mmol), K_2CO_3 (58.3 g, 422 mmol) and CPME (100 ml). Methyl iodide (17.6 ml, 281 mmol) was added slowly and the reaction was agitated at 25° C for 1.5 h. Additional methyl iodide (17.6 ml, 281 mmol) was added slowly and the reaction was agitated at 25° C for an additional 16 h. K_2CO_3 was removed through filtration and the cake was washed with CPME (50 ml). The filtrate was concentrated to an oil that was purified by normal phase column chromatography to give methyl 3-cyclopropyl-2-methyl-3-oxopropanoate **5**[1] in 59% yield.

*(E)-Methyl 3-cyclopropyl-2-methyl-3-(tosyloxy)acrylate **1-E**:*

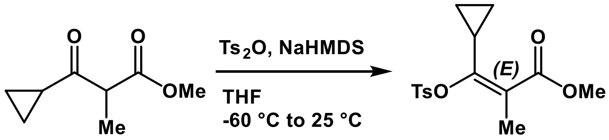

5 **1-*E***

To an oven-dried round-bottomed flask under nitrogen atmosphere was added methyl 3-cyclopropyl-2-methyl-3-oxopropanoate **5** (26.0 g, 166 mmol) and THF (260 ml). The mixture was cooled to -78 °C and 1M NaHMDS solution in THF (200 ml, 200 mmol) was added over 20 min, maintaining the internal temperature below -60 °C, followed by a THF rinse (65 ml). The reaction was agitated at -78 °C for 20 min and Ts_2O (65.2 g, 200 mmol) in THF (520 ml) was added over 20 min, maintaining the internal temperature below -60 °C. The reaction was agitated at -78 °C for 20 min, then warmed to 25 °C and agitated for 16 h. A thick slurry formed. The reaction was quenched with 750 ml of aqueous 0.5M $NaHCO_3$ and the phases were separated. The aqueous layer was back-extracted twice with 250 ml of EtOAc. The organic layers were combined and washed with 500 ml of saturated aqueous NaCl, dried over $MgSO_4$ and filtered. Upon concentration to an oil, the crude product was purified by normal phase column chromatography to give (*E*)-methyl 3-cyclopropyl-2-methyl-3-(tosyloxy)acrylate **1-*E***[1] in 31% yield.

*(E)-Methyl 3-(3-(benzyloxy)phenyl)-3-cyclopropyl-2-methylacrylate **2-E** and (Z)-Methyl 3-(3-(benzyloxy)phenyl)-3-cyclopropyl-2-methylacrylate **2-Z**:*

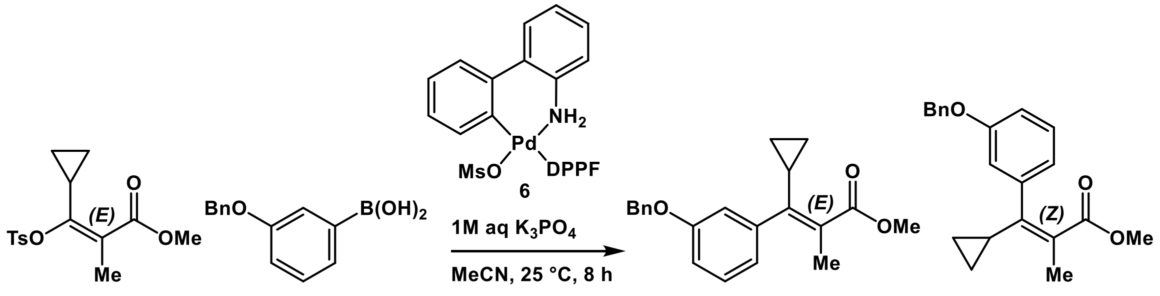

1-*E* **3** **2-*E*** **2-*Z***

To a reaction vial in a positive nitrogen pressure glove box was charged (*E*)-methyl 3-cyclopropyl-2-methyl-3-(tosyloxy)acrylate **1-*E*** (1.00 g, 3.22 mmol), (3-(benzyloxy)phenyl)boronic acid **3** (0.808 g, 3.54 mmol), and palladium precatalyst **6** (0.298 g, 0.322 mmol). Anhydrous ACN (15 ml) and degassed 1M aqueous K_3PO_4 (9.67 ml, 9.67 mmol) were added and the reaction was agitated at 25 °C for 8 h. To the reaction was added 10 ml of saturated aqueous NH_4Cl and the organic layer was separated from the aqueous layer. The aqueous layer was back-extracted twice with 10 ml of EtOAc. The combined organic layers were washed with 10 ml of saturated aqueous NaCl, dried over $MgSO_4$ and filtered. Upon concentration to an oil, the crude product was purified by normal phase column chromatography to give (*E*)-methyl 3-(3-(benzyloxy)phenyl)-3-cyclopropyl-2-methylacrylate **2-*E***[2] in 52% and (*Z*)-methyl 3-(3-(benzyloxy)phenyl)-3-cyclopropyl-2-methylacrylate **2-*Z***[1] in 33% yield.

*(2,6-dimethylphenyl)diphenylphosphane **L20**:*

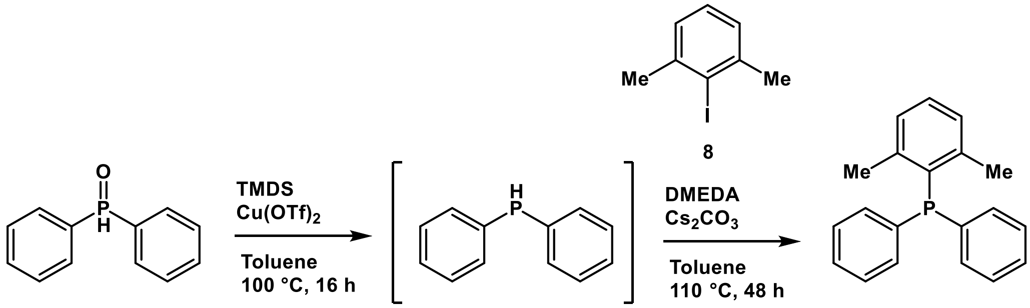

7 **L20**

To a reaction vial in a positive nitrogen pressure glove box was charged $Cu(OTf)_2$ (0.268 g, 0.742 mmol), diphenylphosphine oxide **7** (1.00 g, 4.95 mmol), anhydrous toluene (20 ml) and TMDS (1.76 ml, 9.89 mmol). The reaction was agitated at 100 °C for 5 h with needle venting to avoid pressure build-up. Additional TMDS (0.868 ml, 4.95 mmol) was charged and the reaction was agitated at 100 °C for an additional 16 h with no venting. Upon cooling to 25 °C, to the reaction vial was added DMEDA (0.106 ml, 0.989 mmol), Cs_2CO_3 (3.22 g, 9.89 mmol) and 2-iodo-1,3-dimethylbenzene **8** (1.15 g, 4.95 mmol). The suspension was agitated at 110 °C for 48 h. The reaction was cooled to 0 °C and KOH (20.6 ml, 61.8 mmol) in MeOH (3N) was added slowly to maintain the temperature below 0 °C. The mixture was agitated at 25 °C for 5 h. The quenched mixture was removed from the glovebox and 30 ml water was added. The resulting solution was back-extracted three times with 50 ml EtOAc. The combined organic layers were washed with 50 ml of 1M aqueous HCl, followed by 50 ml of 0.5M aqueous $NaHCO_3$. The organic layer

was decanted to remove elemental copper, dried over Na_2SO_4 and filtered. Upon concentration to an oil, the crude product was purified by normal phase column chromatography to give (2,6-dimethylphenyl)diphenylphosphane **L20**[3] in 13% yield.

3. Data Integration

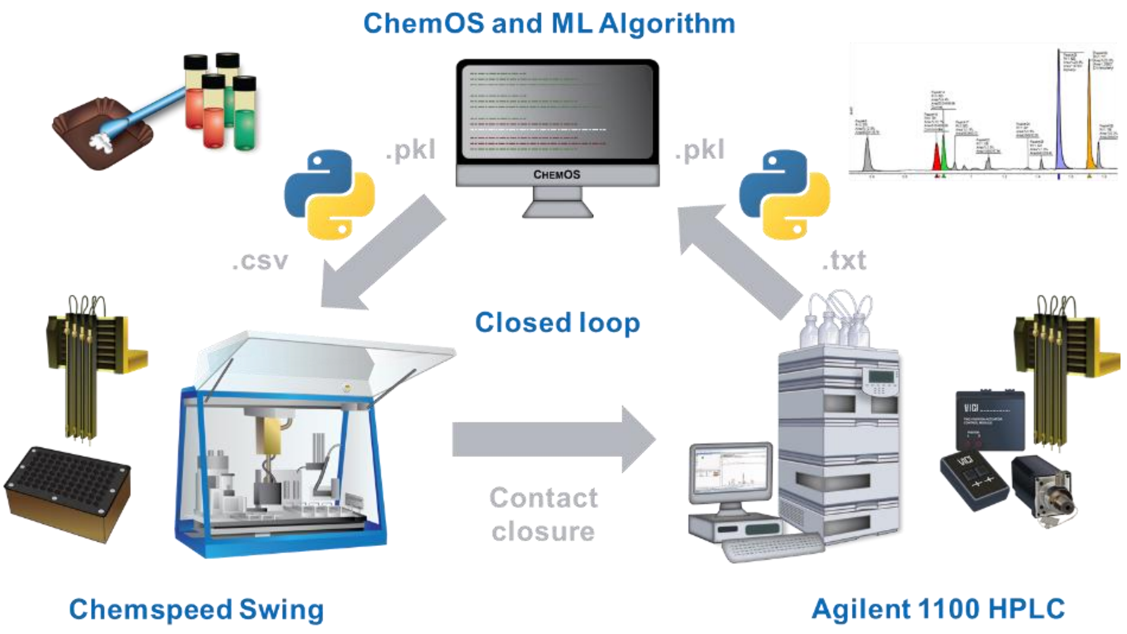

Figure SI-3: Schematic of data integration established through Python.

The Python script used in this work provided an interface between the tools involved in the optimization. This script performed three primary operations: receiving instructions from ChemOS, conveying them to the Chemspeed, monitoring for HPLC results, and conveying those results back to ChemOS. The script has been made publicly available on Gitlab at https://gitlab.com/heingroup/cs-auto-optimization under the MIT open source license.

ChemOS parameters were conveyed to the experimental computer via a file syncing service in the form of pickled dictionaries (dictionaries written to file with Python's pickle standard library). Parameter values were provided in ratios and equivalents, which were converted to volumes for the Chemspeed to dispense based on stored concentrations. Stock mixture concentrations were defined in a separate file, and all dispensing volumes were determined by their relation to the volume and concentration of the vinyl tosylate (held constant for every reaction). For every value, the volume to dispense was determined, rounded to the nearest microliter (the smallest practically accurate volume that the Chemspeed SWING was capable of dispensing), then back-calculated to the actual ratio used. The parameter dictionary was updated with these values so that ChemOS was provided with the true values used to run the experiment. Finally, the total volume was calculated and the total well volume was made up to 200 µL with acetonitrile. Every volume was then written to a csv file which the Chemspeed was programmed to reference for dispensing volumes.

For interpreting and returning experiment result to Chemspeed, the script monitored the sample data directory for the Agilent HPLC. The HPLC method was configured to output a text report after method

completion, which the script parsed and related to the *E*- and *Z*-product peaks by retention time, and a predetermined response factor was used to calculate the assay yield (see SI section 6.2). The result data was then returned to ChemOS via the file syncing service.

4. Autonomous optimization protocol

4.1 Csv file with dispense volumes

A csv file was automatically generated through the Python script as ChemOS instructions became available and referenced in the Chemspeed Autosuite protocol for dispense volumes, with each column being mapped to a distinct protocol source zone. The Python script also recorded analytical results to this csv file as Chemstation report files became available, mainly for recordkeeping purposes. See Supplementary Data 2 for an example CSV file.

4.2 Sample Chemspeed Autosuite protocol

The below Chemspeed Autosuite protocol was executed in campaigns 2 and 3, running autonomously over four days.

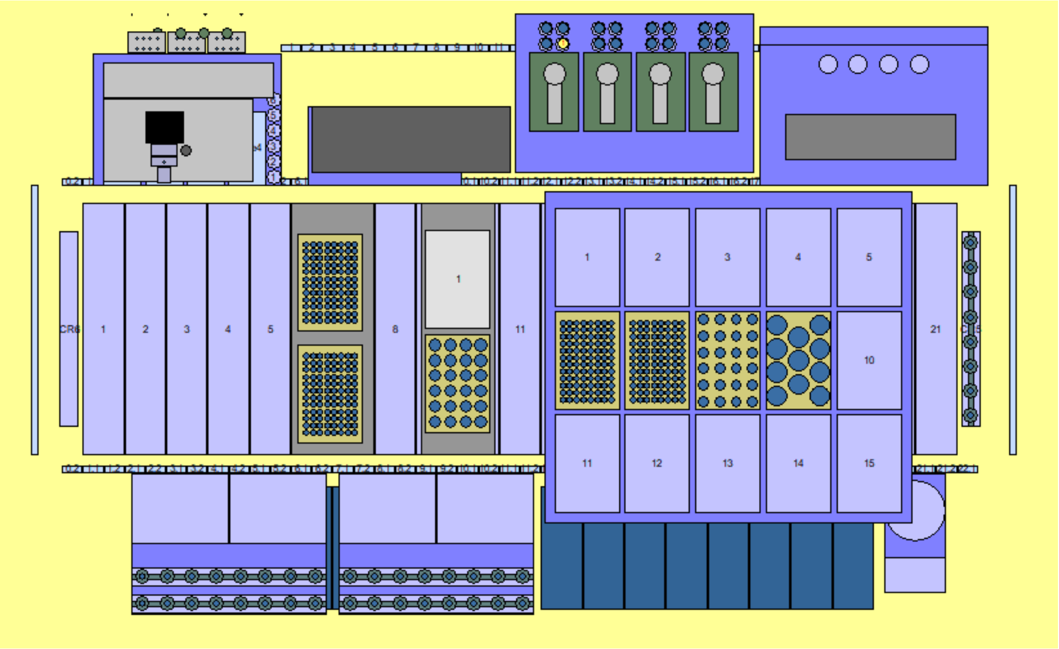

Figure SI-4: Chemspeed deck layout.

1	Transfer Volumetrically	Transfer liquid from valve ports C to Waste 1 with Needle Head #1	
2	Macro Task	Execute Once	
1	Execute Operation	VP Stirrers: Stir	
2	Show Dialog	Input-Dialog for 'reaction_length': 'Please start hplc sequence and enter length	
3	Autonomous Optimization	Execute Once	
1	Set Variable	reaction_time := reaction_length	
2	Log Data	Log ''reaction_time=' + reaction_time + ' ; exit_code=' + exitCode' to category '	
3	Transfer Volumetrically	Transfer liquid from Port C 1 to Injection valve 2 Inject with Needle Head #1	
4	Macro Task	Execute Once	Experimental set-up
1	Macro Task	Execute While 'Experiment_ID <> Experiment_count'	csv import task
1	Wait	Waiting for 5 seconds	
2	Import CSV	import row 'Experiment_count' from file '2019-11-05 chemspeed values.csv''.	
<insert sub tasks here>			
2	Heat / Cool	Thermostat ON on zone Reactions	Temperature
3	Log Data	Log ''Experiment_ID=' + Experiment_ID + ' ; Experiment_count=' + Experiment_	
4	Log Data	Log ''Reaction_starttime=' + Timestamp() + ' ; exit_code=' + exitCode' to categ	
5	Transfer Volumetrically	Transfer liquid from Pd to variable 'one_reaction' with Needle Head #1	Pd transfer
6	Ligands	Execute Once	Ligand transfer
1	Transfer Volumetrically	Transfer liquid from Ligand1 to variable 'one_reaction' with Needle Head #1	
2	Transfer Volumetrically	Transfer liquid from Ligand2 to variable 'one_reaction' with Needle Head #1	
3	Transfer Volumetrically	Transfer liquid from Ligand3 to variable 'one_reaction' with Needle Head #1	
4	Transfer Volumetrically	Transfer liquid from Ligand4 to variable 'one_reaction' with Needle Head #1	
5	Transfer Volumetrically	Transfer liquid from Ligand5 to variable 'one_reaction' with Needle Head #1	
6	Transfer Volumetrically	Transfer liquid from Ligand6 to variable 'one_reaction' with Needle Head #1	
7	Transfer Volumetrically	Transfer liquid from Ligand7 to variable 'one_reaction' with Needle Head #1	
8	Transfer Volumetrically	Transfer liquid from Ligand8 to variable 'one_reaction' with Needle Head #1	
9	Transfer Volumetrically	Transfer liquid from Ligand9 to variable 'one_reaction' with Needle Head #1	
10	Transfer Volumetrically	Transfer liquid from Ligand10 to variable 'one_reaction' with Needle Head #1	
11	Transfer Volumetrically	Transfer liquid from Ligand11 to variable 'one_reaction' with Needle Head #1	
12	Transfer Volumetrically	Transfer liquid from Ligand12 to variable 'one_reaction' with Needle Head #1	
13	Transfer Volumetrically	Transfer liquid from Ligand13 to variable 'one_reaction' with Needle Head #1	
14	Transfer Volumetrically	Transfer liquid from Ligand14 to variable 'one_reaction' with Needle Head #1	
15	Transfer Volumetrically	Transfer liquid from Ligand15 to variable 'one_reaction' with Needle Head #1	
16	Transfer Volumetrically	Transfer liquid from Ligand16 to variable 'one_reaction' with Needle Head #1	
17	Transfer Volumetrically	Transfer liquid from Ligand17 to variable 'one_reaction' with Needle Head #1	
18	Transfer Volumetrically	Transfer liquid from Ligand18 to variable 'one_reaction' with Needle Head #1	
19	Transfer Volumetrically	Transfer liquid from Ligand19 to variable 'one_reaction' with Needle Head #1	
20	Transfer Volumetrically	Transfer liquid from Ligand20 to variable 'one_reaction' with Needle Head #1	
21	Transfer Volumetrically	Transfer liquid from Ligand21 to variable 'one_reaction' with Needle Head #1	
22	Transfer Volumetrically	Transfer liquid from Ligand22 to variable 'one_reaction' with Needle Head #1	
23	Transfer Volumetrically	Transfer liquid from Ligand23 to variable 'one_reaction' with Needle Head #1	
24	Transfer Volumetrically	Transfer liquid from valve ports C to Waste 1 with Needle Head #1	
<insert sub tasks here>			
7	Wait	Waiting for 7:00 minutes	
8	Transfer Volumetrically	Transfer liquid from E-Tosyl_IS to variable 'one_reaction' with Needle Head #1	E-Tosylate transfer
9	Transfer Volumetrically	Transfer liquid from ArBA to variable 'one_reaction' with Needle Head #1	ArBA transfer
10	Transfer Volumetrically	Transfer liquid from ACN Anhydrous to variable 'one_reaction' with Needle He	ACN transfer
11	Transfer Volumetrically	Transfer liquid from valve ports C to Waste 1 with Needle Head #1	
12	Log Data	Log ''PdOAc2_vol=' + Pd_vol + '; E_Tosyl_IS_vol=' + E_Tosyl_IS_vol + '; ArBA_v	
13	Set Variable	reaction_t0 := Timestamp()	
14	Transfer Volumetrically	Transfer liquid from K3PO4 to variable 'one_reaction' with Needle Head #1	K3PO4 transfer
15	Export CSV	'Export in file 'Timestamp.csv''.	
16	Log Data	Log ''Reaction_t0=' + reaction_t0 + '; K3PO4_vol=' + K3PO4_vol + '; exit_code=	
17	Set Variable	Experiment_count := Experiment_count + 1	
18	Set Variable	reaction_count := reaction_count + 1	
<insert sub tasks here>			

5	Macro Task	Execute Once	HPLC sampling
1	HPLC Macro Task	Execute Once	
1	Import CSV	import row 'sample_count' from file 'Timestamp.csv''.	
2	Macro Task	Execute While 'reaction_time >= Timestamp() - reaction_t0'	Time count
1	Set Variable	reaction_runtime := Timestamp() - reaction_t0	
2	Wait	Waiting for 5 seconds	
<insert sub tasks here>			
3	Log Data	Log ''Sample=' + sample_count + '; reaction_runtime=' + reaction_runtime + '	
4	Transfer Volumetrically	Transfer liquid from variable 'one_reaction_hplc' to variable 'one_sample_hplc'	Reaction Sample
5	Transfer Volumetrically	Transfer liquid from variable 'one_sample_hplc' to variable 'one_sample_hplc' v	Sample mixing task
6	Transfer Volumetrically	Transfer liquid from variable 'one_sample_hplc' to Injection valve 2 Load with N	HPLC Sample
7	Wait	Waiting for 2 seconds	
8	Switch Contact	Switch electrical contact 1 on	
9	Wait	Waiting for 2 seconds	
10	Switch Contact	Switch electrical contact 1 off	
11	Transfer Volumetrically	Transfer liquid from Port C 1 to Injection valve 2 Inject with Needle Head #1	
12	Set Variable	sample_count := sample_count + 1	
<insert sub tasks here>			
<insert sub tasks here>			
<insert sub tasks here>			
4	Execute Operation	VP Stirrers: Stir	
5	Heat / Cool	Thermostat OFF on zone Reactions	
<insert sub tasks here>			

5. Machine learning algorithms

The ML algorithms used for experiment planning in this study, Phoenics[4] and Gryffin[5], leverage fundamental concepts from Bayesian optimization in combination with kernel density estimation. Bayesian optimization is an approach to global optimization for applications where the evaluation of a single parameter point is highly time or resource demanding. While several formulations exist, Bayesian optimization follows a two-step strategy to suggest parameter points for future evaluation: (1) constructing a statistical approximation to the considered experiment based on collected measurements, and (2) locating parameter points for which the approximation predicts promising performance. Phoenics and Gryffin construct the statistical approximation based on kernel density estimates of evaluated parameters and suggest promising parameter points with an explicit balance of exploitative and explorative sampling behavior with native support for parallel optimization. To further elaborate, promising parameter points are based on a Bayesian optimization strategy. Instead of Gaussian processes, which are commonly employed in Bayesian optimization frameworks, Gryffin uses Bayesian neural networks. However, Bayesian neural networks are not used to fit experimental measurements. Instead, Gryffin constructs kernel densities of previously sampled experimental parameters from predictions of the Bayesian neural network, which are weighted by the associated measurements to construct a surrogate model (balancing explorative and exploitative sampling strategies via an explicitly chosen sampling parameter) from which future parameters are suggested. The acquisition function explicitly accounts for underexplored regions in the parameter space, such that overfitting is not a concern in the applied framework. Additional details around the BNN model are listed below:

- hidden layers: 3
- nodes per layer: 50
- sampling parameters: equally spaces across the [-1, +1] interval
- no automatic tuning
- initial random seed: 100691

5.1 Process constrained optimization in the context of autonomous experimentation

The autonomous experimentation workflow described in the main text targeted the optimization of both processing conditions and categorical design choices with the goal to maximize the stereoselectivity of a Suzuki-Miyaura coupling reaction. One such reaction can be executed within about two hours on the employed Chemspeed system. However, the Chemspeed system offers the opportunity to run multiple reactions at once. With this parallelization capability, the overall runtime of an experimental campaign consisting of 192 experiments could, in principle, be reduced substantially.

Executing multiple reactions on this system simultaneously introduces a critical limitation to the decision-making process concerning the suggestion of promising reaction parameters: the temperature can only be controlled collectively for all concurrent reactions as opposed to an independent control for all other reaction parameters. This limitation requires adaptations to the decision-making algorithm to account for this process constraint.

For a mathematical formulation of this adaption, we consider a parameter domain X, which we can split into a domain Xc on which parameters are process constrained and a domain Xuc on which parameters are not process constrained, such that $X = X^c \cup X^{uc}$. The basic process constrained batch algorithm implemented in this study is inspired by a previously reported algorithm.[6] Following the aforementioned partitioning of the parameter domain, we first search for promising values for the constrained parameter values $x^c \in X^c$, for which we marginalize the collected feedback onto the X^c domain. Subsequently, we search for promising parameter choices for $x^{uc} \in X^{uc}$ while considering the feedback on the entire domain X given the specific choice of x^c which we determined in the first step. Implementing this process in the kernel-based Bayesian optimization framework provided by Phoenics allows us to choose different sampling policies for the constrained and the unconstrained parameters. In this study, we choose two sampling policies for the constrained parameter and eight policies for the unconstrained parameters to reflect the fact that we executed eight reactions simultaneously.

6. Analytical data

6.1 ^{1}H NMR weight % purity determinations

Vinyl tosylate **1-*E***, coupled product **2-*E***, and coupled product **2-*Z*** were assayed for weight % purity by ^{1}H NMR against an internal standard of known weight and purity (99% 1,3,5-trimethoxybenzene). The tables and figures below show the weight % purity calculations based on ^{1}H NMR spectra using the equation $Purity_{cpd}= Purity_{std}*Area_{cpd}/Area_{std}*Protons_{std}/Protons_{cpd}*MW_{cpd}/MW_{std}*Weight_{std}/Weight_{cpd}$.

Table SI-6: Weight % purity calculation for 1-*E*

	Peak Area (counts)	Protons (#)	MW (g/mol)	Weight (mg)	Purity (wt%)
Internal Std.	5.30	3	168.19	19.75	99%
**1-*E***	2.02	2	310.36	21.41	96%

Table SI-7: Weight % purity calculation for 2-*E*

	Peak Area (counts)	Protons (#)	MW (g/mol)	Weight (mg)	Purity (wt%)
Internal Std.	5.35	3	168.19	19.83	99%
**2-*E***	2.03	2	322.40	22.75	94%

Table SI-8: Weight % purity calculation for 2-*Z*

	Peak Area (counts)	Protons (#)	MW (g/mol)	Weight (mg)	Purity (wt%)
Internal Std.	5.26	3	168.19	17.90	99%
**2-*Z***	1.96	2	322.40	19.55	97%

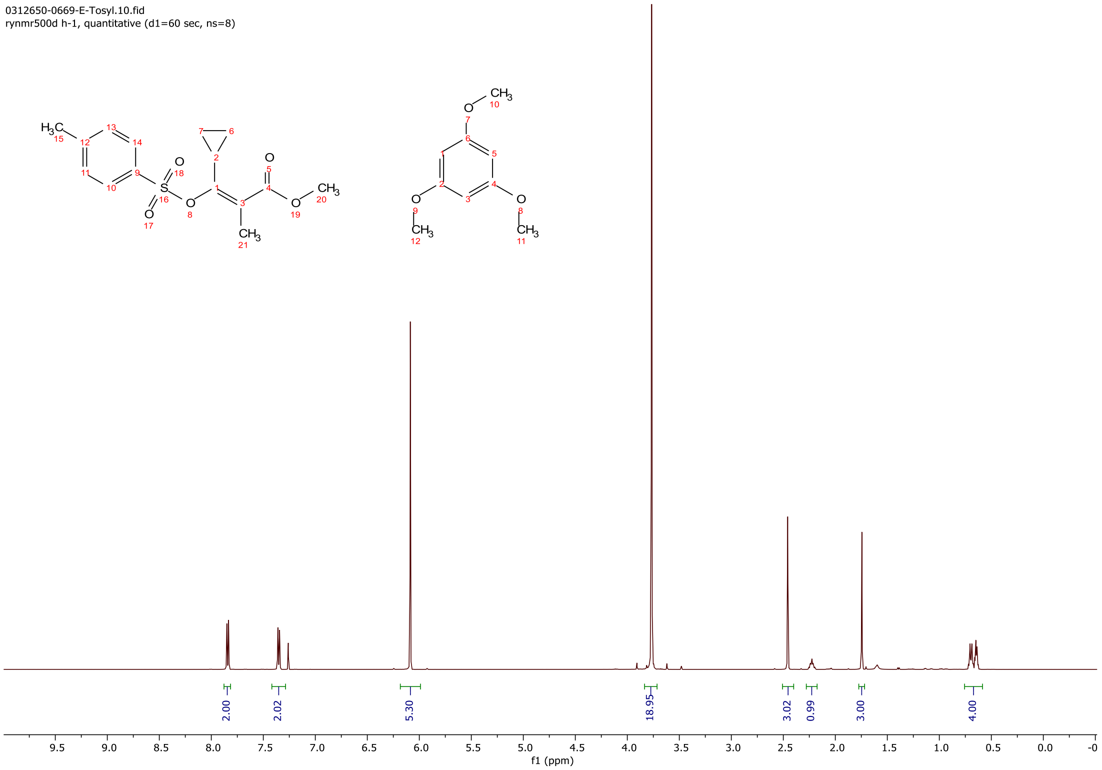

Figure SI-5: ^{1}H NMR for weight % purity determination of vinyl tosylate 1-*E*.

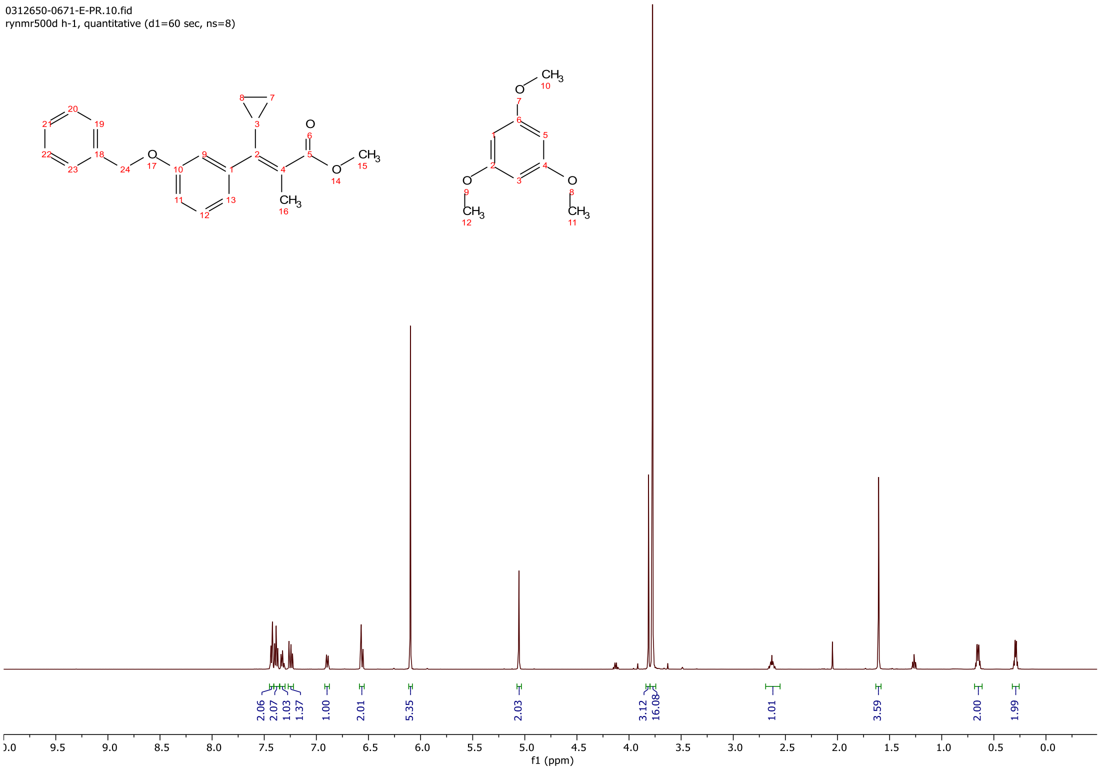

Figure SI-6: ^{1}H NMR for weight % purity determination of Suzuki product 2-*E*.

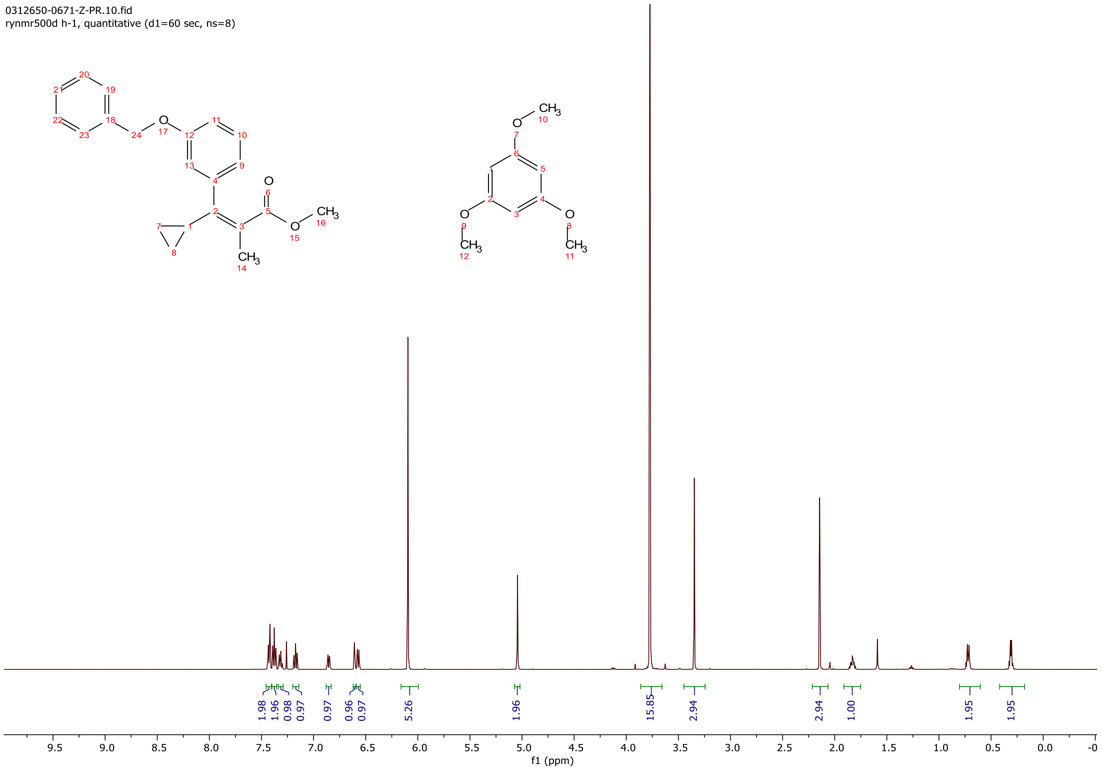

Figure SI-7: ^{1}H NMR for weight % purity determination of Suzuki product 2-*Z*.

6.2 Calibration curves

HPLC-UV calibration curves were generated to determine the mol % distribution of reaction components **1-*E***, **2-*E*** and **2-*Z*** through measuring the ratio of their peak areas to an internal standard (**IS**) peak area (10 mol% 1,3,5-trimethoxybenzene). Four mixtures were prepared containing 10 mol% **IS** and 100, 75, 50 or 25 mol% of each compound. Each mixture was acquired on the Chemspeed online HPLC system at 210 nm in duplicate. The mol% of compound in each mixture was plotted against the measured peak area ratio of each compound to **IS**, generating the calibration curves provided in figure SI-8. Linear regression with the intercept set at zero allowed for the determination of response factors for straightforward conversion of compound to **IS** ratio to mol % compound through the following equation: mol % compound = m * peak area of compound at 210 nm / peak area of **IS** at 210 nm, where m = the response factor determined through calibration. These response factors were included in the Python script for automated calculation of mol % compound from the HPLC data.

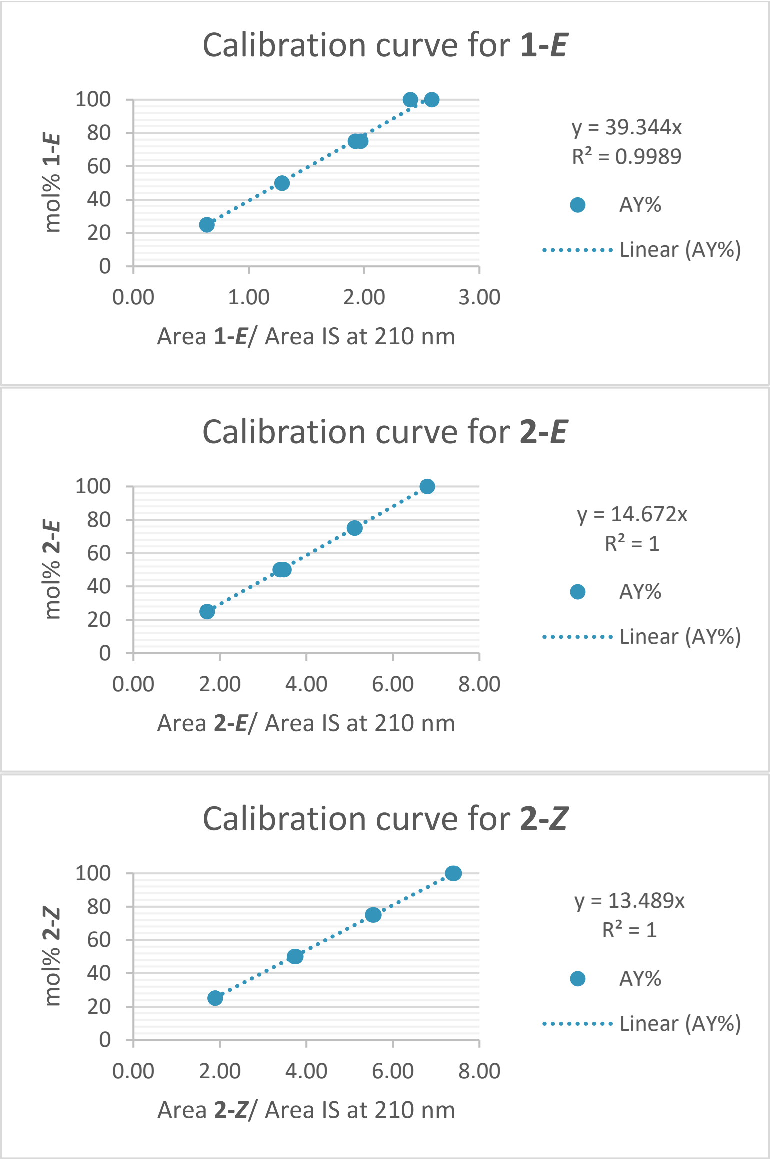

Figure SI-8: Calibration curves for conversion of compound (1-*E*, 2-*E*, 2-*Z*) to internal standard (IS) HPLC area ratios at 210 nm to mol % distribution in the reaction mixture.

6.3 Preliminary data

Table SI-9: Reaction conditions and data included in Figure 1 of the main manuscript

**Expt**	**Temp**	**Pd mol%**	**P ligand**	**P/Pd**	**ArBA eq.**	**1-*E* mol%**	**2-*E* mol%**	**2-*Z* mol%**
1	25.00	4.10	None	0.00	1.95	1	3	91
2	25.00	1.70	L11	2.35	1.05	45	1	48
3	25.00	1.70	L7	2.35	1.05	2	60	35

6.4 Campaign 1 data

Table SI-10: Reaction conditions and data included in Figure 3 of the main manuscript

Expt	Temp	Pd mol%	P ligand	P/Pd	ArBA eq.	1-*E* mol%	2-*E* mol%	2-*Z* mol%
1	29.14	2.50	L5	3.36	1.25	95	1	5
2	29.14	3.90	L2	2.36	1.03	86	3	3
3	29.14	2.80	L1	1.71	1.30	43	25	45
4	29.14	2.70	L11	1.93	1.03	9	2	88
5	29.14	2.00	L8	2.80	1.45	59	3	31
6	29.14	1.20	L8	3.33	1.75	61	2	29
7	29.14	1.90	L4	1.47	1.33	11	10	78
8	29.14	2.90	L3	2.07	1.53	86	0	2
9	25.50	4.90	L3	1.80	1.95	41	4	46
10	25.50	4.90	L12	0.82	1.93	1	2	95
11	25.50	4.90	L7	3.59	1.98	79	1	1
12	25.50	4.00	L7	0.60	1.90	1	26	75
13	25.50	1.30	L10	4.00	1.90	48	1	33
14	25.50	4.90	L10	3.76	1.85	8	2	70
15	25.50	1.80	L1	0.44	1.43	54	26	41
16	25.50	2.50	L1	3.68	1.08	76	2	6
17	10.33	4.80	L12	3.42	1.20	2	3	98
18	10.33	1.40	L1	0.57	1.33	47	23	23
19	10.33	2.50	L2	1.12	2.00	41	26	21
20	10.33	1.00	L6	3.20	1.38	75	0	11
21	10.33	1.40	L10	0.57	1.88	36	1	52
22	10.33	1.90	L1	0.63	1.23	38	26	23
23	10.33	2.40	L1	0.50	1.33	30	4	64
24	10.33	4.60	L6	0.61	1.08	11	2	80
25	13.10	3.60	L5	3.56	1.90	77	0	10
26	13.10	4.80	L9	0.50	1.20	12	5	78
27	13.10	2.80	L1	3.00	1.93	77	1	1
28	13.10	2.20	L5	0.91	1.08	27	2	70
29	13.10	1.90	L1	1.47	1.00	86	6	10
30	13.10	4.90	L3	0.90	1.23	28	5	53
31	13.10	1.70	L2	0.71	1.05	57	17	19
32	13.10	2.70	L12	3.26	1.93	1	3	94
33	39.90	4.50	L7	0.89	1.38	2	6	93
34	39.90	1.10	L4	0.73	1.98	64	1	21
35	39.90	1.90	L9	0.63	2.00	26	2	70
36	39.90	5.00	L10	0.56	1.48	1	3	87
37	39.90	4.10	L9	3.71	1.60	76	3	4

38	39.90	2.40	L12	1.17	1.95	3	2	92
39	39.90	4.90	L4	3.18	1.55	76	1	9
40	39.90	3.50	L1	1.03	2.00	10	35	40
41	12.00	3.50	L9	2.29	1.43	30	8	61
42	12.00	1.90	L11	0.42	1.25	37	1	62
43	12.00	2.20	L7	1.45	1.90	12	32	54
44	12.00	2.80	L3	2.43	1.45	86	0	1
45	12.00	1.90	L4	0.42	1.25	28	2	65
46	12.00	4.80	L5	1.58	1.70	73	0	8
47	12.00	2.10	L1	0.57	1.15	30	28	26
49	39.61	1.20	L2	3.00	1.15	41	33	23
50	39.61	2.70	L6	3.26	1.88	99	1	8
51	39.61	3.90	L3	1.23	1.88	8	7	74
52	39.61	3.90	L7	1.23	1.88	1	47	49
53	39.61	3.90	L1	1.54	2.00	4	32	52
54	39.61	4.10	L4	1.46	1.90	1	13	89
55	39.61	1.10	L10	2.18	1.03	43	1	48
56	39.61	2.30	L11	4.00	1.93	8	3	95
57	39.58	3.90	L1	3.69	1.15	49	6	19
58	39.58	1.30	L11	1.85	1.65	76	1	34
59	39.58	4.30	L7	3.53	1.03	83	1	1
60	39.58	4.60	L7	1.22	1.80	1	31	70
61	39.58	4.30	L10	4.00	1.03	3	1	45
62	39.58	4.10	L7	1.37	1.90	1	49	61
63	39.58	3.40	L7	1.88	1.80	2	51	44
64	39.58	4.60	L7	1.22	1.80	1	38	61
65	24.47	1.00	L6	0.80	1.90	65	0	18
66	24.47	4.60	L4	3.83	1.35	93	0	2
67	24.47	1.00	L3	0.80	1.90	83	0	19
68	24.47	3.40	L2	4.00	1.55	109	0	0
69	24.47	4.60	L6	3.83	1.35	77	0	11
70	24.47	4.00	L7	1.00	1.98	1	41	55
71	24.47	4.60	L11	3.39	1.38	1	3	96
72	24.47	4.50	L8	3.38	1.40	86	1	2
73	35.96	4.20	L11	2.38	1.33	2	2	63
74	35.96	3.20	L7	2.00	1.68	2	48	48
75	35.96	3.80	L5	2.53	1.38	90	1	4
76	35.96	3.10	L8	2.06	1.73	35	5	54
77	35.96	3.50	L4	2.06	1.73	6	11	80
78	35.96	3.00	L1	2.13	1.88	14	24	47
79	35.96	1.70	L8	0.47	1.55	60	1	39
80	35.96	2.30	L12	0.70	1.53	12	2	89
81	30.03	1.20	L4	2.00	2.00	29	7	51

82	30.03	1.20	L7	2.00	2.00	68	12	50
83	30.03	3.40	L7	1.65	1.75	2	45	58
84	30.03	3.90	L3	0.51	1.05	17	5	71
85	30.03	3.80	L7	2.74	1.70	50	17	16
86	30.03	3.80	L5	0.74	1.03	3	3	92
87	30.03	3.90	L11	0.62	1.50	1	3	98
88	30.03	4.80	L10	2.00	1.55	1	3	78
89	36.51	3.00	L7	1.87	2.00	3	46	38
90	36.51	3.80	L5	3.89	1.98	102	1	4
91	36.51	2.80	L7	2.86	1.73	14	45	34
92	36.51	1.60	L3	1.00	1.03	44	3	45
93	36.51	3.00	L7	1.87	2.00	2	59	46
94	36.51	1.30	L12	4.00	1.38	40	2	66
95	36.51	4.60	L3	2.87	1.98	101	0	2
96	36.51	2.30	L7	2.09	1.63	1	52	44
97	12.39	2.90	L12	3.17	1.03	17	2	68
98	12.39	1.30	L5	2.46	2.00	72	0	7
99	12.39	2.40	L4	1.50	1.80	49	5	43
100	12.39	2.90	L7	3.31	1.03	87	0	0
101	12.39	4.50	L4	1.96	1.03	59	4	31
102	12.39	3.50	L6	0.69	1.63	19	2	74
103	12.39	3.10	L4	3.48	1.10	91	0	1
104	12.39	1.30	L7	3.08	1.83	50	28	14
105	35.27	2.10	L7	2.67	1.03	2	40	40
106	35.27	2.00	L1	2.00	1.63	30	20	39
107	35.27	2.80	L3	3.86	1.48	91	0	1
108	35.27	2.20	L4	2.00	1.65	2	13	90
109	35.27	4.80	L8	1.50	1.15	17	6	69
110	35.27	3.30	L12	1.70	2.00	1	4	104
111	35.27	3.00	L11	4.00	1.33	2	3	90
112	35.27	2.80	L5	3.86	1.48	74	0	4
113	23.50	1.00	L12	2.40	1.13	59	0	22
114	23.50	2.80	L11	2.29	1.98	17	3	89
115	23.50	1.10	L10	2.91	1.08	55	1	32
116	23.50	3.70	L7	1.51	1.93	2	56	39
117	23.50	1.10	L2	2.91	1.05	103	0	0
118	23.50	3.70	L7	1.51	1.93	2	65	42
119	23.50	1.10	L7	2.91	1.08	1	57	34
120	23.50	2.30	L6	0.52	1.03	26	2	63

6.5 Campaign 2 data

Table SI-11: Reaction conditions and data included in Figure 5 of the main manuscript

Expt	Temp	Pd mol%	P ligand	P/Pd	1-*E* mol%	2-*E* mol%	2-*Z* mol%
9	32.81	2.70	L31	2.59	85	17	6
10	32.81	1.30	L30	1.69	35	51	20
11	32.81	2.80	L29	2.86	77	1	5
12	32.81	1.80	L22	1.67	55	15	34
13	32.81	3.40	L12	1.88	1	2	99
14	32.81	1.90	L29	3.26	87	0	3
15	32.81	1.90	L13	1.58	74	16	16
16	32.81	2.00	L27	1.60	13	8	85
17	35.15	1.00	L30	4.00	48	45	17
18	35.15	4.80	L20	3.79	30	34	40
19	35.15	4.20	L15	3.90	106	0	0
20	35.15	1.40	L30	0.57	20	38	48
21	35.15	5.00	L16	3.68	104	1	1
22	35.15	4.30	L30	0.60	1	58	46
23	35.15	1.30	L30	2.00	27	57	22
24	35.15	5.00	L26	3.92	1	51	54
25	10.37	1.00	L4	0.60	43	2	67
26	10.37	1.00	L32	0.60	93	6	10
27	10.37	4.70	L2	2.43	107	1	0
28	10.37	1.20	L21	4.00	108	1	0
29	10.37	1.80	L18	3.89	106	0	0
30	10.37	4.80	L17	1.88	88	9	9
31	10.37	4.80	L24	1.17	69	18	17
32	10.37	5.00	L19	1.44	69	8	30
33	36.37	4.50	L30	0.76	4	63	41
34	36.37	5.00	L25	2.32	4	30	65
35	36.37	5.00	L19	2.32	108	0	0
36	36.37	1.40	L30	2.86	28	57	22
37	36.37	3.30	L25	0.55	8	34	67
38	36.37	1.30	L30	2.62	25	60	23
39	36.37	4.90	L14	0.94	1	3	101
40	36.37	1.60	L30	2.63	27	58	22
41	14.32	4.50	L23	3.78	109	3	3
42	14.32	3.00	L14	1.27	7	3	98
43	14.32	2.30	L24	2.17	108	0	0
44	14.32	2.70	L27	1.63	42	5	62
45	14.32	4.40	L16	1.14	11	6	89

46	14.32	3.80	L32	3.95	106	0	0
47	14.32	2.60	L28	2.08	106	0	1
48	14.32	3.20	L29	1.06	67	1	24
49	37.96	1.10	L30	2.73	36	54	21
50	37.96	1.10	L2	2.91	75	20	12
51	37.96	1.10	L21	2.73	84	7	15
52	37.96	1.10	L24	2.91	102	1	2
53	37.96	1.00	L30	2.60	36	51	19
54	37.96	1.10	L4	2.91	64	5	35
55	37.96	2.20	L17	0.55	4	10	92
56	37.96	1.10	L14	2.73	29	2	77
57	22.52	1.10	L14	3.09	32	2	84
58	22.52	1.10	L30	0.91	55	37	17
59	22.52	4.60	L28	0.83	28	2	76
60	22.52	1.00	L19	2.80	107	0	0
61	22.52	1.20	L12	3.17	14	2	91
62	22.52	4.10	L30	0.59	5	56	43
63	22.52	1.10	L25	3.09	107	0	1
64	22.52	1.00	L30	2.80	61	34	13
65	25.98	4.70	L30	0.85	9	65	33
66	25.98	4.60	L18	2.43	100	0	4
67	25.98	4.20	L4	3.14	105	0	3
68	25.98	2.80	L28	3.93	105	1	1
69	25.98	4.10	L13	3.17	106	0	0
70	25.98	3.10	L32	4.00	103	2	2
71	25.98	4.20	L22	3.24	99	1	5
72	25.98	3.70	L30	0.76	9	62	34
73	27.45	4.70	L30	0.81	7	63	37
74	27.45	3.30	L21	0.97	38	39	26
75	27.45	3.40	L12	0.53	4	3	103
76	27.45	3.40	L18	0.65	19	16	70
77	27.45	3.20	L32	0.81	48	28	29
78	27.45	3.40	L4	0.76	10	9	85
79	27.45	5.00	L26	1.08	1	41	62
80	27.45	5.00	L23	1.04	77	4	27
81	27.40	4.20	L30	1.38	5	72	30
82	27.40	1.40	L19	0.57	13	10	85
83	27.40	3.60	L30	0.94	12	64	29
84	27.40	3.30	L25	1.94	38	23	40
85	27.40	1.70	L29	0.59	16	2	82
86	27.40	1.70	L16	0.59	19	5	84
87	27.40	4.80	L22	0.75	33	27	44
88	27.40	3.60	L31	2.39	75	20	8

89	10.11	4.60	L24	0.87	66	22	19
90	10.11	1.00	L30	2.20	87	14	5
91	10.11	3.10	L15	2.97	108	0	0
92	10.11	3.10	L31	3.16	106	2	1
93	10.11	1.10	L31	2.73	107	1	0
94	10.11	1.00	L13	1.80	93	5	9
95	10.11	1.00	L20	1.80	98	4	5
96	10.11	3.60	L30	3.06	74	21	9
105	22.38	2.50	L16	2.72	42	7	60
106	22.38	1.40	L22	1.71	75	11	19
107	22.38	3.80	L30	0.74	16	55	34
108	22.38	4.80	L30	0.71	8	59	37
109	22.38	4.70	L30	1.62	28	55	22
110	22.38	2.00	L20	1.70	26	35	45
111	22.38	1.30	L23	1.69	105	2	4
112	22.38	4.70	L30	1.57	26	57	23
113	27.86	4.20	L30	1.52	16	64	26
114	27.86	1.70	L32	3.88	103	2	2
115	27.86	1.70	L26	3.88	30	38	39
116	27.86	1.60	L17	3.88	108	0	0
117	27.86	4.40	L30	1.59	11	67	27
118	27.86	3.90	L30	1.69	13	66	26
119	27.86	1.60	L19	4.00	107	0	0
120	27.86	4.00	L30	1.50	13	66	27
121	39.07	4.10	L19	0.54	3	28	76
122	39.07	4.80	L30	0.63	1	61	43
123	39.07	4.40	L30	0.68	1	64	40
124	39.07	3.30	L20	3.94	31	33	40
125	39.07	2.60	L31	4.00	71	23	7
126	39.07	2.60	L26	3.85	9	46	50
127	39.07	2.90	L16	3.93	106	0	0
128	39.07	2.70	L2	4.00	100	5	3
129	31.77	4.10	L30	2.05	10	69	28
130	31.77	4.40	L21	1.86	18	46	41
131	31.77	4.80	L29	3.08	59	0	4
132	31.77	4.20	L30	1.95	7	69	28
133	31.77	4.10	L30	2.05	11	67	27
134	31.77	4.10	L30	2.05	8	69	28
135	31.77	4.80	L23	3.08	99	3	6
136	31.77	4.10	L30	1.90	6	72	29
137	17.56	4.90	L30	2.08	49	41	17
138	17.56	4.70	L30	0.51	22	46	37
139	17.56	4.00	L13	2.05	99	5	3

140	17.56	3.70	L25	2.59	75	12	18
141	17.56	1.90	L24	0.63	47	30	30
142	17.56	1.90	L15	0.53	21	14	71
143	17.56	3.80	L23	2.00	99	3	5
144	17.56	1.90	L25	0.63	38	18	52
145	33.85	4.10	L12	2.83	1	3	98
146	33.85	4.20	L30	1.81	4	71	29
147	33.85	3.80	L30	1.74	7	68	27
148	33.85	3.80	L13	2.63	107	0	0
149	33.85	3.90	L4	3.13	100	1	6
150	33.85	3.60	L4	3.22	100	1	7
151	33.85	3.90	L28	3.13	98	4	2
152	33.85	4.50	L30	1.87	1	73	30
153	16.67	2.80	L22	4.00	109	0	0
154	16.67	5.00	L14	3.12	10	3	93
155	16.67	2.80	L4	4.00	106	0	1
156	16.67	4.90	L15	0.65	13	28	64
157	16.67	2.80	L23	3.86	104	2	3
158	16.67	4.20	L30	0.57	22	50	33
159	16.67	5.00	L28	3.04	106	0	0
160	16.67	5.00	L27	3.24	49	10	59
161	35.05	3.80	L30	2.42	6	73	29
162	35.05	4.50	L30	2.31	3	72	29
163	35.05	3.80	L30	1.21	3	73	31
164	35.05	4.50	L30	2.31	1	73	30
165	35.05	4.00	L31	1.05	40	19	42
166	35.05	4.20	L30	1.81	2	72	29
167	35.05	1.70	L17	2.47	107	0	0
168	35.05	1.70	L23	2.24	102	2	4
169	11.26	4.20	L13	0.52	35	15	58
170	11.26	2.00	L30	2.90	82	18	7
171	11.26	1.90	L2	1.05	67	25	14
172	11.26	3.90	L18	1.49	72	10	24
173	11.26	3.90	L26	1.49	16	42	48
174	11.26	2.00	L30	2.90	83	18	7
175	11.26	2.20	L12	2.91	7	2	96
176	11.26	4.60	L30	0.61	33	46	27
177	29.84	1.10	L4	2.55	87	3	20
178	29.84	4.10	L2	1.22	30	45	31
179	29.84	3.90	L27	1.49	6	10	89
180	29.84	1.10	L18	2.55	104	0	2
181	29.84	1.90	L21	2.32	98	3	5
182	29.84	4.20	L16	1.48	14	9	80

183	29.84	1.80	L15	2.44	56	13	37
184	29.84	3.80	L30	1.74	13	68	28
185	21.92	3.20	L26	2.06	7	49	53
186	21.92	4.30	L30	0.98	19	59	25
187	21.92	3.00	L17	1.40	72	12	22
188	21.92	3.00	L12	1.40	1	3	99
189	21.92	3.00	L31	1.40	48	9	50
190	21.92	4.60	L13	1.43	51	24	28
191	21.92	2.60	L22	1.00	63	16	25
192	21.92	4.80	L27	1.38	13	8	85

6.6 Campaign 3 data

Campaign 3 incorporated 15 manually selected computed phosphine descriptors into the algorithmic optimization to relate ligands among one another with respect to steric and electronic features. see Supplementary Data 1 for a list of the selected descriptors.

Table SI-12: Reaction conditions and data included in Figure SI-9

Expt	Temp	Pd mol%	P ligand	P/Pd	1-*E* mol%	2-*E* mol%	2-*Z* mol%
9	32.81	2.00	L27	1.60	12	6	86
10	32.81	1.90	L29	3.26	69	1	7
11	32.81	2.80	L29	2.86	66	1	7
12	32.81	2.70	L31	2.59	63	23	8
13	32.81	1.30	L30	1.69	23	56	24
14	32.81	1.90	L13	1.58	46	23	29
15	32.81	1.80	L22	1.67	40	18	12
16	32.81	3.40	L12	1.88	6	3	92
17	27.45	4.90	L32	3.10	87	8	7
18	27.45	1.00	L31	1.20	41	5	56
19	27.45	4.80	L14	3.83	1	3	94
20	27.45	1.10	L4	0.73	10	3	88
21	27.45	1.20	L24	0.67	26	33	44
22	27.45	4.80	L16	0.83	1	8	86
23	27.45	2.10	L27	0.86	2	4	95
24	27.45	1.10	L19	0.73	13	13	74
25	10.15	1.50	L26	3.87	35	36	35
26	10.15	2.20	L12	3.45	3	5	93
27	10.15	4.80	L31	1.25	60	7	33
28	10.15	1.40	L19	2.57	105	0	0
29	10.15	4.30	L14	0.51	6	3	92
30	10.15	2.20	L16	3.82	46	5	50

31	10.15	4.70	L28	0.64	11	2	88
32	10.15	1.50	L13	3.73	101	0	0
33	31.07	1.30	L27	2.31	16	6	82
34	31.07	3.40	L16	3.29	36	8	55
35	31.07	3.50	L26	3.94	1	45	54
36	31.07	1.10	L20	2.00	11	50	51
37	31.07	3.30	L14	3.82	6	3	91
38	31.07	1.30	L2	2.15	34	39	26
39	31.07	1.00	L4	2.00	24	9	69
40	31.07	1.10	L19	2.18	53	17	30
41	11.12	5.00	L12	3.56	3	3	95
42	11.12	1.80	L23	4.00	97	2	4
43	11.12	4.70	L16	3.02	28	6	66
44	11.12	1.50	L16	0.67	21	3	77
45	11.12	1.50	L30	4.00	79	16	6
46	11.12	2.00	L26	0.60	22	24	55
47	11.12	2.00	L14	0.70	8	3	90
48	11.12	1.70	L26	3.88	28	37	36
49	28.03	2.70	L30	3.70	20	65	23
50	28.03	3.20	L26	3.56	1	47	53
51	28.03	3.40	L16	0.88	2	16	87
52	28.03	2.80	L28	3.71	97	2	1
53	28.03	3.50	L14	0.57	1	3	96
54	28.03	4.00	L26	3.95	1	46	54
55	28.03	3.50	L14	3.89	5	13	90
56	28.03	4.00	L28	3.95	97	2	1
57	15.81	3.60	L26	2.33	5	44	54
58	15.81	3.00	L16	2.07	14	11	78
59	15.81	1.20	L14	3.67	17	2	82
60	15.81	3.10	L31	2.00	37	5	59
61	15.81	1.20	L23	3.67	95	2	4
62	15.81	1.90	L28	3.89	101	0	0
63	15.81	3.20	L17	2.19	80	9	11
64	15.81	3.20	L13	2.06	64	17	17
65	23.80	3.40	L30	3.53	27	54	22
66	23.80	4.20	L28	3.86	101	1	1
67	23.80	4.80	L26	1.67	1	39	59
68	23.80	1.90	L27	3.68	30	12	64
69	23.80	2.00	L29	3.80	77	2	6
70	23.80	2.30	L22	3.74	65	8	25
71	23.80	4.90	L16	2.29	8	8	81
72	23.80	2.30	L16	2.35	25	7	68
73	38.76	4.90	L16	3.71	28	12	59

74	38.76	3.50	L26	3.89	2	44	54
75	38.76	4.80	L17	1.71	24	35	39
76	38.76	3.60	L31	3.94	51	30	10
77	38.76	4.90	L13	1.59	11	41	43
78	38.76	4.90	L23	1.06	43	4	52
79	38.76	3.50	L28	3.54	86	7	2
80	38.76	1.10	L2	1.27	13	49	37
81	21.11	3.20	L32	3.81	96	1	1
82	21.11	3.00	L16	1.07	18	6	77
83	21.11	3.30	L13	3.64	102	0	0
84	21.11	3.50	L22	3.31	49	13	35
85	21.11	3.30	L27	3.64	27	8	72
86	21.11	3.30	L19	3.64	103	0	0
87	21.11	3.20	L4	3.69	99	1	2
88	21.11	1.00	L16	2.40	45	5	53
89	38.19	1.10	L2	0.73	11	48	43
90	38.19	3.00	L16	0.73	1	10	86
91	38.19	1.40	L27	0.86	3	4	87
92	38.19	1.10	L19	0.55	6	2	91
93	38.19	1.00	L32	1.40	43	29	27
94	38.19	1.00	L31	1.40	57	8	36
95	38.19	3.00	L12	0.73	5	3	94
96	38.19	2.90	L14	0.97	2	3	97
105	10.72	3.20	L24	0.88	50	29	24
106	10.72	3.60	L23	3.78	97	2	4
107	10.72	4.00	L30	2.70	59	30	12
108	10.72	3.20	L16	0.50	16	3	79
109	10.72	3.80	L28	2.89	101	0	0
110	10.72	3.30	L14	1.15	8	2	90
111	10.72	3.80	L14	2.89	16	2	82
112	10.72	3.80	L26	2.16	14	41	47
113	29.63	2.90	L16	1.59	59	12	35
114	29.63	2.60	L14	2.15	6	3	89
115	29.63	2.40	L27	3.83	13	10	80
116	29.63	4.00	L13	2.65	100	0	0
117	29.63	3.40	L26	3.65	1	46	54
118	29.63	2.40	L32	3.83	93	11	2
119	29.63	2.40	L22	3.67	40	13	43
120	29.63	3.50	L28	3.37	96	2	1
121	15.13	3.90	L30	2.36	48	38	15
122	15.13	3.50	L26	2.80	8	45	49
123	15.13	1.40	L26	1.57	24	38	42
124	15.13	4.00	L28	2.50	100	3	0

125	15.13	1.10	L16	1.45	36	3	65
126	15.13	1.40	L24	1.57	77	13	12
127	15.13	1.30	L28	1.38	100	1	2
128	15.13	3.80	L14	2.53	6	3	91
129	34.30	4.20	L23	0.52	2	4	93
130	34.30	4.20	L14	0.57	6	3	93
131	34.30	5.00	L16	2.36	12	11	72
132	34.30	1.20	L22	1.67	49	18	12
133	34.30	4.00	L26	0.70	1	29	70
134	34.30	4.20	L28	3.86	90	7	2
135	34.30	4.20	L26	3.81	0	44	55
136	34.30	3.70	L23	3.89	94	5	5
137	14.31	2.30	L13	2.70	99	2	1
138	14.31	3.50	L28	3.03	101	0	0
139	14.31	3.40	L26	2.76	10	43	48
140	14.31	2.10	L23	2.38	95	4	5
141	14.31	2.30	L28	2.43	104	0	1
142	14.31	3.30	L31	3.09	84	9	11
143	14.31	3.40	L12	3.18	1	2	94
144	14.31	2.20	L14	2.64	11	2	86
145	15.40	5.00	L13	2.00	49	25	25
146	15.40	3.60	L14	2.67	5	3	90
147	15.40	3.30	L30	2.73	52	35	14
148	15.40	2.60	L26	1.31	10	39	52
149	15.40	3.40	L26	2.94	4	48	51
150	15.40	2.30	L23	1.30	76	12	23
151	15.40	3.90	L16	1.38	16	5	78
152	15.40	3.80	L28	2.47	100	1	0
153	39.60	2.80	L28	3.93	90	6	3
154	39.60	3.50	L23	3.94	93	3	6
155	39.60	1.50	L16	2.80	64	8	27
156	39.60	3.40	L16	2.41	30	12	57
157	39.60	1.10	L13	3.82	102	0	0
158	39.60	1.30	L26	3.85	11	42	48
159	39.60	2.90	L30	4.00	2	74	28
160	39.60	3.30	L26	3.82	1	45	54
161	27.71	3.00	L14	3.73	10	4	85
162	27.71	3.20	L23	3.75	93	3	5
163	27.71	3.10	L26	3.48	1	46	53
164	27.71	3.30	L31	3.70	91	17	3
165	27.71	4.10	L32	1.56	49	24	25
166	27.71	4.10	L17	1.46	44	20	33
167	27.71	1.20	L23	3.33	96	2	4

168	27.71	4.10	L13	1.51	32	30	37
169	23.43	3.70	L4	3.68	97	1	4
170	23.43	3.50	L28	1.94	95	2	1
171	23.43	3.30	L26	2.36	0	45	54
172	23.43	3.20	L14	2.31	4	4	92
173	23.43	2.60	L23	1.62	92	3	7
174	23.43	5.00	L30	1.60	11	61	27
175	23.43	3.80	L24	2.05	46	23	29
176	23.43	3.70	L25	3.68	88	3	7
177	18.35	4.90	L18	1.06	26	15	57
178	18.35	5.00	L27	1.12	4	5	88
179	18.35	4.70	L23	2.94	94	2	5
180	18.35	5.00	L22	1.04	45	20	33
181	18.35	5.00	L13	0.84	14	25	57
182	18.35	3.90	L26	0.51	5	24	70
183	18.35	4.80	L19	0.96	10	26	64
184	18.35	4.90	L22	1.06	47	20	32
185	21.54	4.70	L22	1.53	46	19	33
186	21.54	4.70	L22	1.53	46	19	33
187	21.54	1.60	L32	1.13	69	15	18
188	21.54	4.60	L18	1.26	28	17	54
189	21.54	4.70	L22	1.53	42	19	35
190	21.54	4.70	L22	1.53	45	19	33
191	21.54	1.30	L16	1.38	24	4	71
192	21.54	1.80	L26	0.78	10	32	60

6.7 Campaign 3 visualization

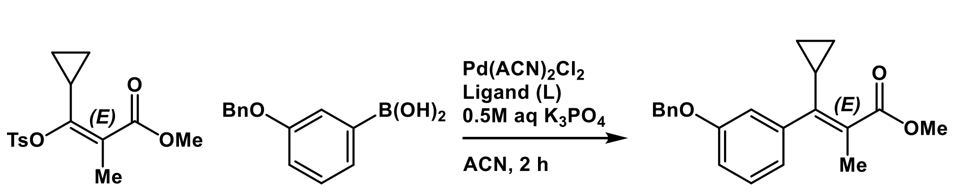

1-*E* **3** **2-*E*** **2-*Z***

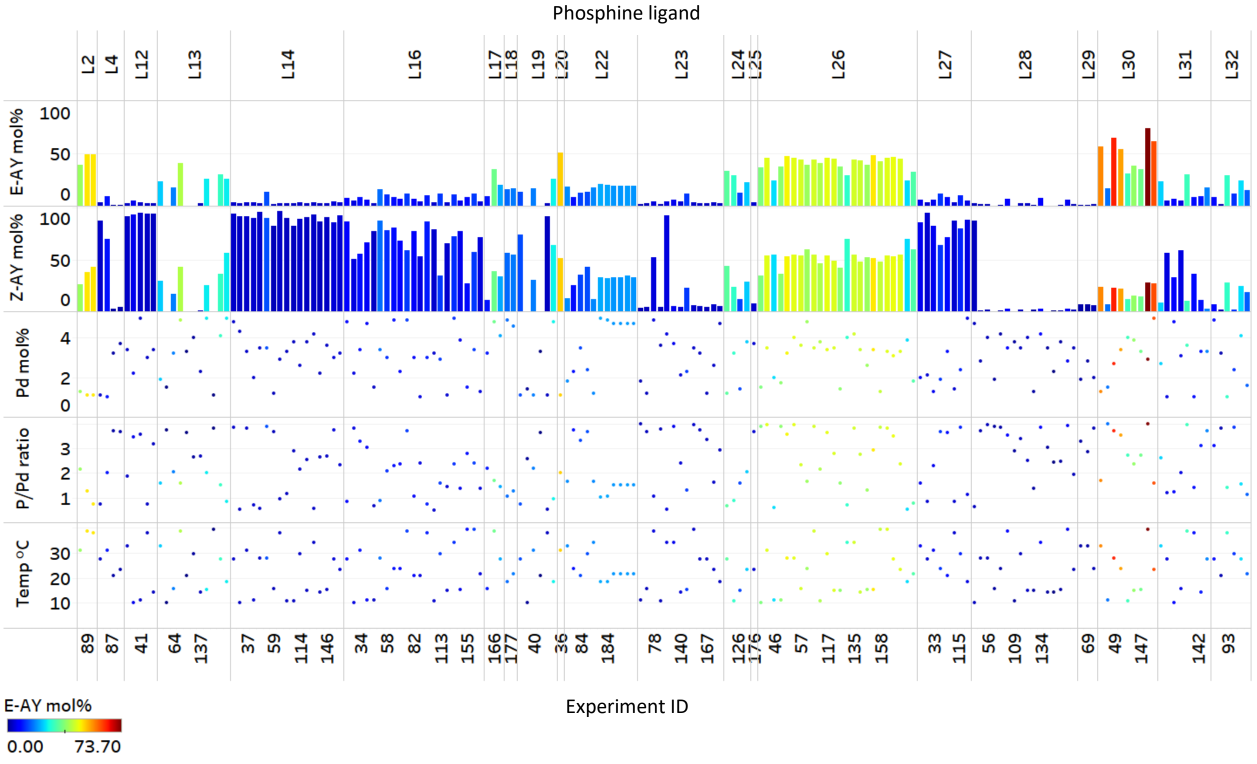

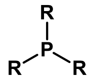

L13: R = Et
L14: R = Mesityl
L15: R = 3,5-Me-4-OMe-Ph
L16: R = nOctyl
L17: R = nPr

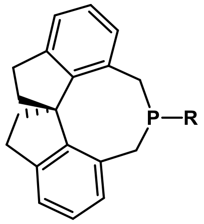

L18: (R)SITCP R = Ph

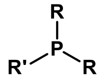

L19: R = Cy R'=Ph
L20: R = Ph R'=2,6-Me-Ph
L21: R = Ph R'=2-OMe-Ph
L22: R = Ph R' = Allyl
L23: R = C_6F_5 R'=Ph
L24: PhAtaPhos R = Ph R'= 4-NMe_2-Ph
L25: R = Ph R'= $(CH_2)_3Si(OEt)_3$
L26: R = 4-(2,2-CF_3-F_7pent)-Ph R'=Ph
L27: m-CroPhos R = tBu R'= *E*-butene

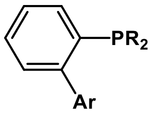

L28: R = Cy Ar = 2,4,6-OMe-Ph
L29: JohnPhos R = tBu Ar = Ph
L30: PhSPhos R= Ph Ar = 2,6-OMe-Ph
L31: SPhos R= Cy Ar = 2,6-OMe-Ph
L32: Et-PhenCarPhos R = Et Ar = Cz

Figure SI-9. Parameters and results of optimization with ligands selected through descriptor clustering with 15 selected phosphine descriptors included in algorithmic optimization (campaign 3). Conditions: 10 µmol **1-*E***, 1 µmol 1,3,5-trimethoxybenzene, 15 µmol **3**, 0.1 - 0.5 µmol $Pd(ACN)_2Cl_2$, 0.05 - 2 µmol **L**, 30 µmol K_3PO_4 (0.5M aq) in ACN (0.05M), 2 h at 10 - 40 °C. Tabulated results are provided in Supplementary Information Table SI-12.

6.8 Standard experiments

In campaigns involving 192 iterations, two sets of eight standard experiments (conditions provided in Figure SI-10) were carried out in in each reaction block in order to measure reproducibility and system performance. We determined that the relative standard deviation (RSD) of measured **2-*E*** and **2-*Z*** yields fell between 6-8 %.

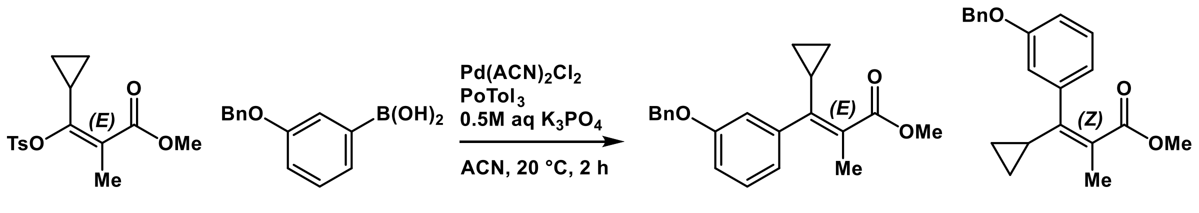

Figure SI-10: Standard experimental conditions.
Conditions: 10 µmol **1-*E***, 1 µmol 1,3,5-trimethoxybenzene, 15 µmol **3**, 0.3 µmol $Pd(ACN)_2Cl_2$, 0.4 µmol **L2** ($PoTol_3$), 30 µmol K_3PO_4 (0.5M aq) in ACN (0.05M), 2 h at 20 °C.

Table SI-13: Standard experiments from blocks 1 and 2 of campaign 2

Expt	Temp	Pd mol%	P ligand	P/Pd	2-*E* mol%	2-*Z* mol%
1	20	2.5	L2	1.52	34	22
2	20	2.5	L2	1.52	32	20
3	20	2.5	L2	1.52	28	17
4	20	2.5	L2	1.52	31	19
5	20	2.5	L2	1.52	30	19
6	20	2.5	L2	1.52	28	17
7	20	2.5	L2	1.52	30	18
8	20	2.5	L2	1.52	31	19
**Average**					**30**	**19**
**SD**					**2**	**1**
**RSD%**					**6**	**8**
97	20	2.5	L2	1.52	28	17
98	20	2.5	L2	1.52	31	18
99	20	2.5	L2	1.52	30	18
100	20	2.5	L2	1.52	28	17
101	20	2.5	L2	1.52	25	15
102	20	2.5	L2	1.52	27	16
103	20	2.5	L2	1.52	28	17
104	20	2.5	L2	1.52	28	18
**Average**					**28**	**17**
**SD**					**2**	**1**
**RSD%**					**6**	**6**

7. Descriptor computation

Detailed electronic and steric descriptor calculations for 365 commercially available phosphine ligands have been disclosed previously.[7]

The main steps are:

- conformational sampling of each ligand using Crest at the GFN2-xTB level in two model states: free ligand, $[LNi(CO)_3]$ complex[8]
- selection of a representative set of conformers based on steric properties as relative energies
- DFT computations for the selected conformers in Gaussian 16 rev C.01:[9]
    - geometry optimization and frequency calculation at the PBE-D3(BJ)/6-31+G(d) level
    - single points for electronic properties at the PBE0-D3(BJ)/def2-TZVP level, including a population analysis with NBO 7.0.7,[10] SMD solvation single point with the preset parameters for chloroform, and single points for the radical cation and radical anion
- reading steric and electronic properties from the DFT computations

The following descriptors (118 total) were used in the subsequent analysis.

Table SI-14: Only Boltzmann-weighted average (suffix _boltz)

descriptor	brief description
vmin_vmin	Vmin = minimum of molecular electrostatic potential in P-lone pair
vmin_r	distance of Vmin from P
fmo_e_homo	HOMO energy
fmo_e_lumo	LUMO energy
fmo_mu	chemical potential = average of HOMO/LUMO energies
fmo_eta	softness = difference of HOMO/LUMO energies
fmo_omega	Global electrophilicity parameter ω
nbo_P	Phosphorus natural population (NBO)
nmr_P	isotropic chemical shielding constant P
nmrtens_sxx_P	anisotropic shielding tensor XX-eigenvalue P
nmrtens_syy_P	anisotropic shielding tensor YY-eigenvalue P
nmrtens_szz_P	anisotropic shielding tensor ZZ-eigenvalue P
efg_amp_P	Electric field gradient tensor amplitude P
efgtens_xx_P	Electric field gradient tensor XX-eigenvalue P
efgtens_yy_P	Electric field gradient tensor YY-eigenvalue P
efgtens_zz_P	Electric field gradient tensor ZZ-eigenvalue P
nuesp_P	Electrostatic potential at P
nbo_lp_P_percent_s	s-orbital percentage of P lone pair (NBO)
nbo_lp_P_occ	P lone pair occupancy (NBO)
nbo_lp_P_e	P lone pair energy (NBO)
nbo_bd_e_max	P-C bonding orbital energy, highest (NBO)
nbo_bd_e_avg	P-C bonding orbital energy, average (NBO)
nbo_bds_e_min	P-C antibonding orbital energy, lowest (NBO)
nbo_bds_e_avg	P-C antibonding orbital energy, average (NBO)

nbo_bd_occ_min	P-C bonding orbital occupancy, lowest (NBO)
nbo_bd_occ_avg	P-C bonding orbital occupancy, average (NBO)
nbo_bds_occ_max	P-C antibonding orbital occupancy, highest (NBO)
nbo_bds_occ_avg	P-C antibonding orbital occupancy, average (NBO)
E_solv_total	Energy of solvation
E_solv_cds	Electrostatic component of energy of solvation
E_solv_elstat	Non-electrostatic component of energy of solvation
fukui_m	Atom-condensed Fukui index f–
fukui_p	Atom-condensed Fukui index f+
vbur_ratio_vbur_vtot	Ratio buried-to-total volume

Table SI-15: All "condensed properties": Boltzmann-weighted average (_boltz), minimum (_min) and maximum (_max) across all conformers, difference between the conformer minimum and maximum (_delta)

descriptor	brief description
dipolemoment	Dipole moment
qpole_amp	Quadrupole moment amplitude
qpoletens_xx	Quadrupole moment tensor XX component
qpoletens_yy	Quadrupole moment tensor YY component
qpoletens_zz	Quadrupole moment tensor ZZ component
pyr_P	Pyramidalization
pyr_alpha	Pyramidalization angle alpha
vbur_vbur	buried volume
vbur_vtot	total volume
vbur_qvbur_min	buried quadrant volume, lowest
vbur_qvbur_max	buried quadrant volume, highest
vbur_qvtot_min	total quadrant volume, lowest
vbur_qvtot_max	total quadrant volume, highest
vbur_max_delta_qvbur	largest volume difference of neighborisc quadrants, buried
vbur_max_delta_qvtot	largest volume difference of neighborisc quadrants, total
sterimol_B1	Sterimol B1
sterimol_B5	Sterimol B5
sterimol_L	Sterimol L
sterimol_burB1	buried Sterimol B1
sterimol_burB5	buried Sterimol B5
sterimol_burL	buried Sterimol L

8. Training set selection

The 118-dimensional descriptor space was transformed using principal component analysis in scikit-learn, retaining the first 4 principal components. The resulting 4-dimensional space was divided into 24 regions by k-means clustering. Candidates for the training set were considered by increasing distance to each

cluster center, taking practical considerations such as availability of the candidates into account. More aspects considering this workflow will be disclosed subsequently.[11]

9. Predictive modeling

Suggestions for E-selective ligands were obtained by two different approaches:

- regression modeling
- classification

Experimental data from campaigns 2 and 3 were used for this procedure. In order to account for the subsequent changes to various reaction parameters during the autonomous optimization runs, the natural log of the highest yield of **2-*E*** that was obtained with each ligand *under any reaction conditions* in all experiments was used as optimization target. This is a practical approximation that is limited by the number of experiments that each ligand was used in and how optimal the other reaction conditions were for each ligand in any experiment.

9.1 Regression modeling

A model ensemble was generated consisting of three sparse linear models (four features each), identified by forward stepwise feature selection as described previously.[12] After feature selection, each model was refit using ridge regression, automatically selecting alpha using internal cross-validation (RidgeCV in scikit-learn). The three models were chosen by diversity of the features and their validation statistics from a 75:25 training:test split determined by the Kennard-Stone algorithm based on the ligand descriptors. The models were then applied to the descriptors of ca. 900 P-based ligands to obtain predicted *max(yield 2-E)* of each model. Ligand candidates were selected based on the variance and average of predictions of each model in the ensemble, as well as practical considerations. Sometimes that entailed changing to a similar but commercially available ligand.

Model 1:

Ridge alpha = 0.001

Features:
p-value   coefficient
1.32E-10   -1.7133 +
2.73E-04   -2.6077 * x5 fmo_mu_boltz
1.34E-03   -2.1383 * x7 fmo_omega_boltz
1.32E-05   1.0014 * x30 nbo_bds_e_min_boltz
3.35E-04   -0.7124 * x98 vbur_qvtot_min_boltz

Training R2 = 0.844
Training Q2 = 0.747
Training MAE = 0.200
Training K-fold R2 = 0.711 (+/- 0.005)

Test R2 = 0.958
Test MAE = 0.169

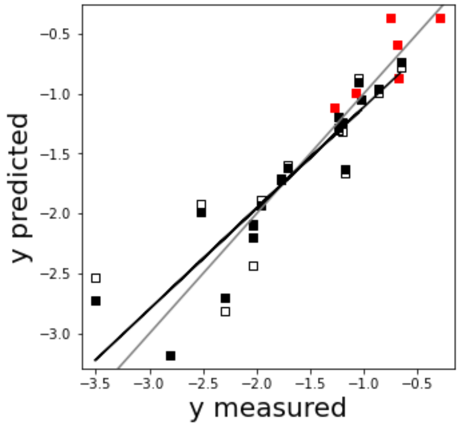

Figure SI-11: Model 1.
y is ln(highest yield of **2-*E***) per ligand in any experiment.

Model 2:

Ridge alpha = 1.0

Features:
p-value coefficient
6.96E-10 -1.7133 +
3.79E-03 0.3742 * x30 nbo_bds_e_min_boltz
5.62E-05 0.5771 * x69 pyr_P_min
7.01E-02 0.1831 * x86 vbur_vtot_delta
4.50E-02 -0.2260 * x160 sterimol_B1_max

Training R2 = 0.784
Training Q2 = 0.673
Training MAE = 0.273
Training K-fold R2 = 0.613 (+/- 0.006)

Test R2 = 0.812
Test MAE = 0.398

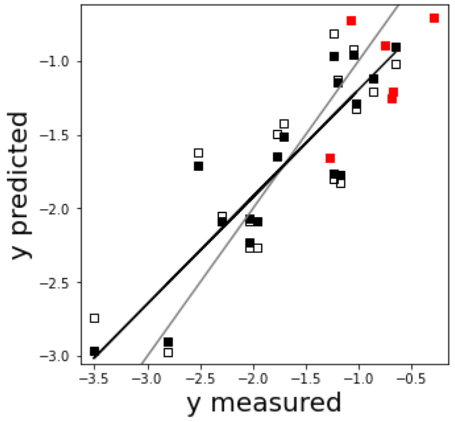

Figure SI-12: Model 2.
y is ln(highest yield of **2-*E***) per ligand in any experiment.

Model 3:

Ridge alpha = 0.1

Features:
p-value coefficient
2.92E-10 -1.7133 +
1.22E-03 0.4852 * x30 nbo_bds_e_min_boltz
1.27E-02 -0.4867 * x51 qpole_amp_delta
3.31E-05 -0.7806 * x75 pyr_alpha_max
9.73E-03 0.3891 * x86 vbur_vtot_delta

Training R2 = 0.820
Training Q2 = 0.708
Training MAE = 0.263
Training K-fold R2 = 0.648 (+/- 0.011)

Test R2 = 0.778
Test MAE = 0.364

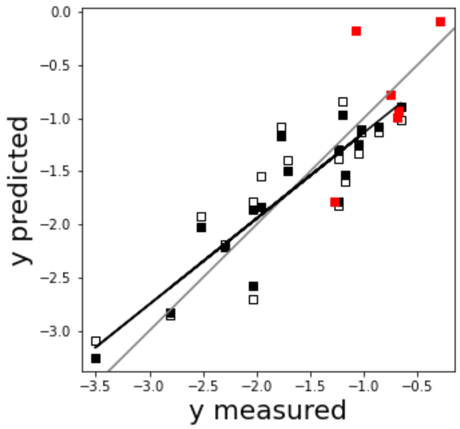

Figure SI-13: Model 3.
y is ln(highest yield of **2-*E***) per ligand in any experiment.

9.2 <u>Classification suggestion</u>

Candidates were selected by their similarity to the most selective ligand (PhSPhos) in the reduced chemical space representation. More details for this procedure will be disclosed subsequently.[11] This procedure entails:

- assign ligands to the classes most selective (PhSPhos) and less selective (all others) based on their experimental results
- train k-nearest neighbors (k = 1) classifier on the 4-dimensional PC space (see above) and apply that classifier to ca. 900 other P-based ligands for an estimate of the "most selective" region in this chemical space representation.
- select ligand candidates that were classified "most selective" based on availability

9.3 Prediction results

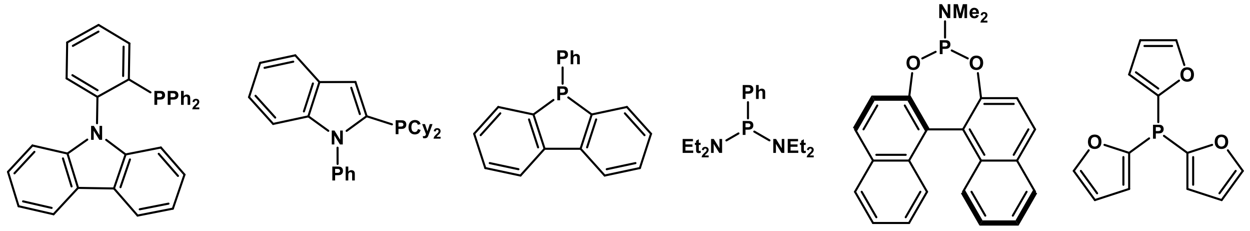

L33: Ph-PhenCarPhos L34: CataCXiumPInCy L35: PhDBP L36: $PhP(NEt_2)_2$ L37: (R)-MonoPhos L38: $P(2\text{-furyl})_3$

Figure SI-14: Selected ligands.

Ligands **L36** and **L38** were selected as commercially available alternatives to the related compounds shown below. The diazaphosphinane predictions were higher average (83% **2-*E***) and lower variance (0.136) than the commercially available ligand L36. The regression predictions for the ligands proposed by classification are shown for comparison. The model predictions for both furyl phosphines had high variances, normally precluding the ligands as candidates, but the simple structure of L38 warranted further investigation nonetheless.

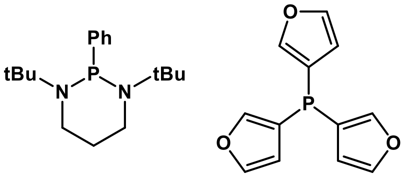

Figure SI-15: Compounds related to selected ligands.

The two ligands with the biggest discrepancy between prediction and experimental results are **L37** and **L38**. The training data did not contain information for heteroatom-substituted phosphorus compounds such as the phosphoramidite **L37**, so a poor prediction is not surprising. As discussed above, **L38** showed a large variance between the predictions, indicating an unsafe prediction that would not normally be taken into consideration, so a large discrepancy is also not unexpected.

Table SI-16: Comparison of predicted vs. experimentally determined yields of product 2-*E*

	name	source	%**2-*E*** pred, mean	ln(%**2-*E***) pred, variance	%**2-*E***, exp
L33	Ph-PhenCarPhos	Classification	22	0.001	39
L34	cataCXiumPInCy	Classification	13	0.039	13
L35	PhDBP	Regression	74	0.136	52
L36	$PhP(NEt_2)_2$	R.+Similarity	60	0.241	53
L37	(R)-MonoPhos	Regression	68	0.040	12
L38	$P(2\text{-furyl})_3$	R.+Similarity	62	0.270	23

[1] This compound was previously characterized in Christensen, M. *et al.* Enantioselective synthesis of α-methyl-β-cyclopropyldihydrocinnamates. *J. Org. Chem.* **81,** (2016).
[2] This compound was previously characterized in Christensen, M. *et al.* Development of an automated kinetic profiling system with online HPLC for reaction optimization. *React. Chem. Eng.* **4,** 1555–1558 (2019).
[3] This compound was previously characterized in Li, Y., Das, S., Zhou, S., Junge, K. & Beller, M. General and selective copper-catalyzed reduction of tertiary and secondary phosphine oxides: Convenient synthesis of phosphines. *J. Am. Chem. Soc.* **134,** 9727–9732 (2012).
[4] Häse, F., Roch, L. M., Kreisbeck, C. & Aspuru-Guzik, A. Phoenics: A Bayesian Optimizer for Chemistry. *ACS Cent. Sci.* **4,** 1134–1145 (2018).
[5] Häse, F., Roch, L. M. & Aspuru-Guzik, A. Gryffin: An algorithm for Bayesian optimization for categorical variables informed by physical intuition with applications to chemistry. (2020).
[6] Algorithm 1 reported in Vellanki, P. *et al.* Process-constrained batch Bayesian Optimisation. (2017).
[7] Gensch, T. et al. A Comprehensive Discovery Platform for Organophosphorus Ligands for Catalysis. Preprint at https://doi.org/10.26434/chemrxiv.12996665.v1 (2021).
[8] Pracht, P., Bohle, F. & Grimme, S. Automated exploration of the low-energy chemical space with fast quantum chemical methods. *Phys. Chem. Chem. Phys.* **22,** 7169–7192 (2020); Bannwarth, C., Ehlert, S. & Grimme, S. GFN2-xTB - An Accurate and Broadly Parametrized Self-Consistent Tight-Binding Quantum Chemical Method with Multipole Electrostatics and Density-Dependent Dispersion Contributions. *J. Chem. Theory Comput.* **15,** 1652–1671 (2019).
[9] Gaussian 16, Revision C.01, M. J. Frisch, G. W. Trucks, H. B. Schlegel, G. E. Scuseria, M. A. Robb, J. R. Cheeseman, G. Scalmani, V. Barone, G. A. Petersson, H. Nakatsuji, X. Li, M. Caricato, A. V. Marenich, J. Bloino, B. G. Janesko, R. Gomperts, B. Mennucci, H. P. Hratchian, J. V. Ortiz, A. F. Izmaylov, J. L. Sonnenberg, D. Williams-Young, F. Ding, F. Lipparini, F. Egidi, J. Goings, B. Peng, A. Petrone, T. Henderson, D. Ranasinghe, V. G. Zakrzewski, J. Gao, N. Rega, G. Zheng, W. Liang, M. Hada, M. Ehara, K. Toyota, R. Fukuda, J. Hasegawa, M. Ishida, T. Nakajima, Y. Honda, O. Kitao, H. Nakai, T. Vreven, K. Throssell, J. A. Montgomery, Jr., J. E. Peralta, F. Ogliaro, M. J. Bearpark, J. J. Heyd, E. N. Brothers, K. N. Kudin, V. N. Staroverov, T. A. Keith, R. Kobayashi, J. Normand, K. Raghavachari, A. P. Rendell, J. C. Burant, S. S. Iyengar, J. Tomasi, M. Cossi, J. M. Millam, M. Klene, C. Adamo, R. Cammi, J. W. Ochterski, R. L. Martin, K. Morokuma, O. Farkas, J. B. Foresman, and D. J. Fox, Gaussian, Inc., Wallingford CT, 2019.
[10] E. D. Glendening, J. K. Badenhoop, A. E. Reed, J. E. Carpenter, J. A. Bohmann, C. M. Morales, P. Karafiloglou, C. R. Landis, and F. Weinhold, Theoretical Chemistry Institute, University of Wisconsin, Madison, WI (2018)
[11] Gensch, *et. al.* Design and Application of a Training Set for Monophosphine Ligands in Metal Catalysis. Preprint will be available at DOI: 10.26434/chemrxiv.13160939 (2020).
[12] Guo, J. Y., Minko, Y., Santiago, C. B. & Sigman, M. S. Developing Comprehensive Computational Parameter Sets to Describe the Performance of Pyridine-Oxazoline and Related Ligands. *ACS Catal.* **7,** 4144–4151 (2017).
