# Supplementary material for: Data-science driven autonomous process optimization
Source: Commun Chem. 2021 Aug 2;4:112. doi: 10.1038/s42004-021-00550-x (PMC9814253; doi:10.1038/s42004-021-00550-x)
Supplement: Supplementary file 2 — Description of Additional Supplementary Files [file 42004_2021_550_MOESM2_ESM.pdf]

Description of Additional Supplementary Files

File Name: Supplementary Data 1

Description: Computed phosphine features utilized in the third optimization campaign.

File Name: Supplementary Data 2

Description: Chemspeed dispense volumes automatically generated through Python for autonomous dispensing.

File Name: Supplementary Movie 1

Description: Short recording of the Chemspeed robot deck during experimental execution.

File Name: Supplementary Movie 2

Description: Animated chart of 2-E yield over time in the second optimization campaign. Time is represented by Experiment ID. Color is correlated to reaction temperature and size is correlated to phosphine to palladium ratio.
